# Neurologic complications in patients receiving aortic versus subclavian versus femoral arterial cannulation for post-cardiotomy extracorporeal life support: results of the PELS observational multicenter study

**DOI:** 10.1186/s13054-024-05047-2

**Published:** 2024-08-07

**Authors:** Giovanni Chiarini, Silvia Mariani, Anne-Kristin Schaefer, Bas C. T. van Bussel, Michele Di Mauro, Dominik Wiedemann, Diyar Saeed, Matteo Pozzi, Luca Botta, Udo Boeken, Robertas Samalavicius, Karl Bounader, Xiaotong Hou, Jeroen J. H. Bunge, Hergen Buscher, Leonardo Salazar, Bart Meyns, Daniel Herr, Sacha Matteucci, Sandro Sponga, Kollengode Ramanathan, Claudio Russo, Francesco Formica, Pranya Sakiyalak, Antonio Fiore, Daniele Camboni, Giuseppe Maria Raffa, Rodrigo Diaz, I-wen Wang, Jae-Seung Jung, Jan Belohlavek, Vin Pellegrino, Giacomo Bianchi, Matteo Pettinari, Alessandro Barbone, José P. Garcia, Kiran Shekar, Glenn J. R. Whitman, Roberto Lorusso, on behalf of the PELS Investigators, Samuel Heuts, Luca Conci, Jawad Khalil, Sven Lehmann, Jean-Francois Obadia, Antonio Loforte, Davide Pacini, Nikolaos Kalampokas, Agne Jankuviene, Karl Bounader, Erwan Flecher, Dinis Dos Reis Miranda, Kogulan Sriranjan, Michael A. Mazzeffi, Marco Di Eusanio, Igor Vendramin, Graeme MacLaren, Vitaly Sorokin, Alessandro Costetti, Chistof Schmid, Roberto Castillo, Tomas Grus, Marco Solinas

**Affiliations:** 1 Cardio-Thoracic Surgery Department, Cardiovascular Research Institute Maastricht, Maastricht University Medical Centre, P. Debyelaan, 25-6202AZ Maastricht, The Netherlands; 2 Intensive Care Unit, Spedali Civili University Hospital, Brescia, Italy; 3 Cardiac Surgery Unit, Fondazione IRCCS San Gerardo dei Tintori, Monza, Italy; 4 Department of Cardiac Surgery, Medical University of Vienna, Vienna, Austria; 5 Department of Intensive Care Medicine, and Cardiovascular Research Institute Maastricht, Maastricht, The Netherlands; 6 Department of Cardiac Surgery, Karl Landsteiner University, University Clinic St, Pölten, St. Pölten, Austria; 7 Department of Cardiac Surgery, Leipzig Heart Center, Leipzig, Germany; 8 Department of Cardiac Surgery, Louis Pradel Cardiologic Hospital, Lyon, France; 9 Division of Cardiac Surgery, IRCCS Azienda Ospedaliero-Universitaria Di Bologna, Bologna, Italy; 10 Department of Cardiac Surgery, Medical Faculty, Heinrich Heine University, Duesseldorf, Germany; 11 II Department of Anesthesiology, Centre of Anesthesia, Intensive Care and Pain Management, Vilnius University Hospital Santariskiu Klinikos, Vilnius, Lithuania; 12 Division of Cardiothoracic and Vascular Surgery, Pontchaillou University Hospital, Rennes, France; 13 Center for Cardiac Intensive Care, Beijing Institute of Heart, Lung, and Blood Vessels Diseases, Beijing Anzhen Hospital, Capital Medical University, Beijing, China; 14 Department of Intensive Care Adults and Cardiology, Erasmus MC, Rotterdam, The Netherlands; 15 Department of Intensive Care Medicine, Center of Applied Medical Research, St Vincent’s Hospital, Darlinghurs, NSW Australia; 16 University of New South Wales, Sydney, Australia; 17 Department of Cardiology, Fundación Cardiovascular de Colombia, Bucaramanga, Colombia; 18 Department of Cardiac Surgery, Department of Cardiovascular Sciences, University of Leuven, Louvain, Belgium; 19 Departments of Medicine and Surgery, University of Maryland, Baltimore, USA; 20 SOD Cardiochirurgia Ospedali Riuniti ‘Umberto I - Lancisi – Salesi’ Università Politecnica delle Marche, Ancona, Italy; 21 Division of Cardiac Surgery, Cardiothoracic Department, University Hospital of Udine, Udine, Italy; 22 Cardiothoracic Intensive Care Unit, National University Heart Centre, National University Hospital, Singapore, Singapore; 23 Cardiac Surgery Unit, Cardiac Thoracic and Vascular Department, Niguarda Hospital, Milan, Italy; 24 Department of Medicine and Surgery, Cardiac Surgery Unit, University of Parma, University Hospital of Parma, Parma, Italy; 25 Division of Cardiovascular and Thoracic Surgery, Department of Surgery, Faculty of Medicine Siriraj Hospital, Mahidol University, Bangkok, Thailand; 26 Department of Cardiac Surgery, Hôpitaux Universitaires Henri Mondor, Assistance Publique-Hôpitaux de Paris, Creteil, France; 27 Department of Cardiothoracic Surgery, University Medical Center Regensburg, Regensburg, Germany; 28 Department for the Treatment and Study of Cardiothoracic Diseases and Cardiothoracic Transplantation, IRCCS-ISMETT (Istituto Mediterraneo Per I Trapianti e Terapie Ad Alta Specializzazione), Palermo, Italy; 29 Departamento de Anestesia, ECMO Unit, Clínica Las Condes, Las Condes, Santiago, Chile; 30 Division of Cardiac Surgery, Memorial Healthcare System, Hollywood, FL 33021 USA; 31 Department of Thoracic and Cardiovascular Surgery, Korea University Anam Hospital, Seoul, South Korea; 32 2nd Department of Internal Medicine, Cardiovascular Medicine General Teaching Hospital and 1st Faculty of Medicine, Charles University in Prague, Prague, Czech Republic; 33 Intensive Care Unit, The Alfred Hospital, Melbourne, VIC Australia; 34 Ospedale del Cuore Fondazione Toscana “G. Monasterio”, Massa, Italy; 35 Department of Cardiovascular Surgery, Ziekenhuis Oost-Limburg, Genk, Belgium; 36 Cardiac Surgery Unit, IRCCS Humanitas Research Hospital, Rozzano, MI Italy; 37 IU Health Advanced Heart and Lung Care, Indiana University Methodist Hospital, Indianapolis, IN USA; 38 Adult Intensive Care Services, The Prince Charles Hospital, Brisbane, Australia; 39 Cardiac Intensive Care Unit, Johns Hopkins Hospital, Baltimore, MD USA

**Keywords:** Extracorporeal membrane oxygenation, Cardiac surgery, Neurologic complications, Cardiac arrest, Stroke, ICH

## Abstract

**Background:**

Cerebral perfusion may change depending on arterial cannulation site and may affect the incidence of neurologic adverse events in post-cardiotomy extracorporeal life support (ECLS). The current study compares patients' neurologic outcomes with three commonly used arterial cannulation strategies (aortic vs. subclavian/axillary vs. femoral artery) to evaluate if each ECLS configuration is associated with different rates of neurologic complications.

**Methods:**

This retrospective, multicenter (34 centers), observational study included adults requiring post-cardiotomy ECLS between January 2000 and December 2020 present in the Post-Cardiotomy Extracorporeal Life Support (PELS) Study database. Patients with Aortic, Subclavian/Axillary and Femoral cannulation were compared on the incidence of a composite neurological end-point (ischemic stroke, cerebral hemorrhage, brain edema). Secondary outcomes were overall in-hospital mortality, neurologic complications as cause of in-hospital death, and post-operative minor neurologic complications (seizures). Association between cannulation and neurological outcomes were investigated through linear mixed-effects models.

**Results:**

This study included 1897 patients comprising 26.5% Aortic (n = 503), 20.9% Subclavian/Axillary (n = 397) and 52.6% Femoral (n = 997) cannulations. The Subclavian/Axillary group featured a more frequent history of hypertension, smoking, diabetes, previous myocardial infarction, dialysis, peripheral artery disease and previous stroke. Neuro-monitoring was used infrequently in all groups. Major neurologic complications were more frequent in Subclavian/Axillary (Aortic: n = 79, 15.8%; Subclavian/Axillary: n = 78, 19.6%; Femoral: n = 118, 11.9%; *p* < 0.001) also after mixed-effects model adjustment (OR 1.53 [95% CI 1.02–2.31], *p* = 0.041). Seizures were more common in Subclavian/Axillary (n = 13, 3.4%) than Aortic (n = 9, 1.8%) and Femoral cannulation (n = 12, 1.3%, *p* = 0.036). In-hospital mortality was higher after Aortic cannulation (Aortic: n = 344, 68.4%, Subclavian/Axillary: n = 223, 56.2%, Femoral: n = 587, 58.9%, *p* < 0.001), as shown by Kaplan–Meier curves. Anyhow, neurologic cause of death (Aortic: n = 12, 3.9%, Subclavian/Axillary: n = 14, 6.6%, Femoral: n = 28, 5.0%, *p* = 0.433) was similar.

**Conclusions:**

In this analysis of the PELS Study, Subclavian/Axillary cannulation was associated with higher rates of major neurologic complications and seizures. In-hospital mortality was higher after Aortic cannulation, despite no significant differences in incidence of neurological cause of death in these patients. These results encourage vigilance for neurologic complications and neuromonitoring use in patients on ECLS, especially with Subclavian/Axillary cannulation.

**Graphical abstract:**

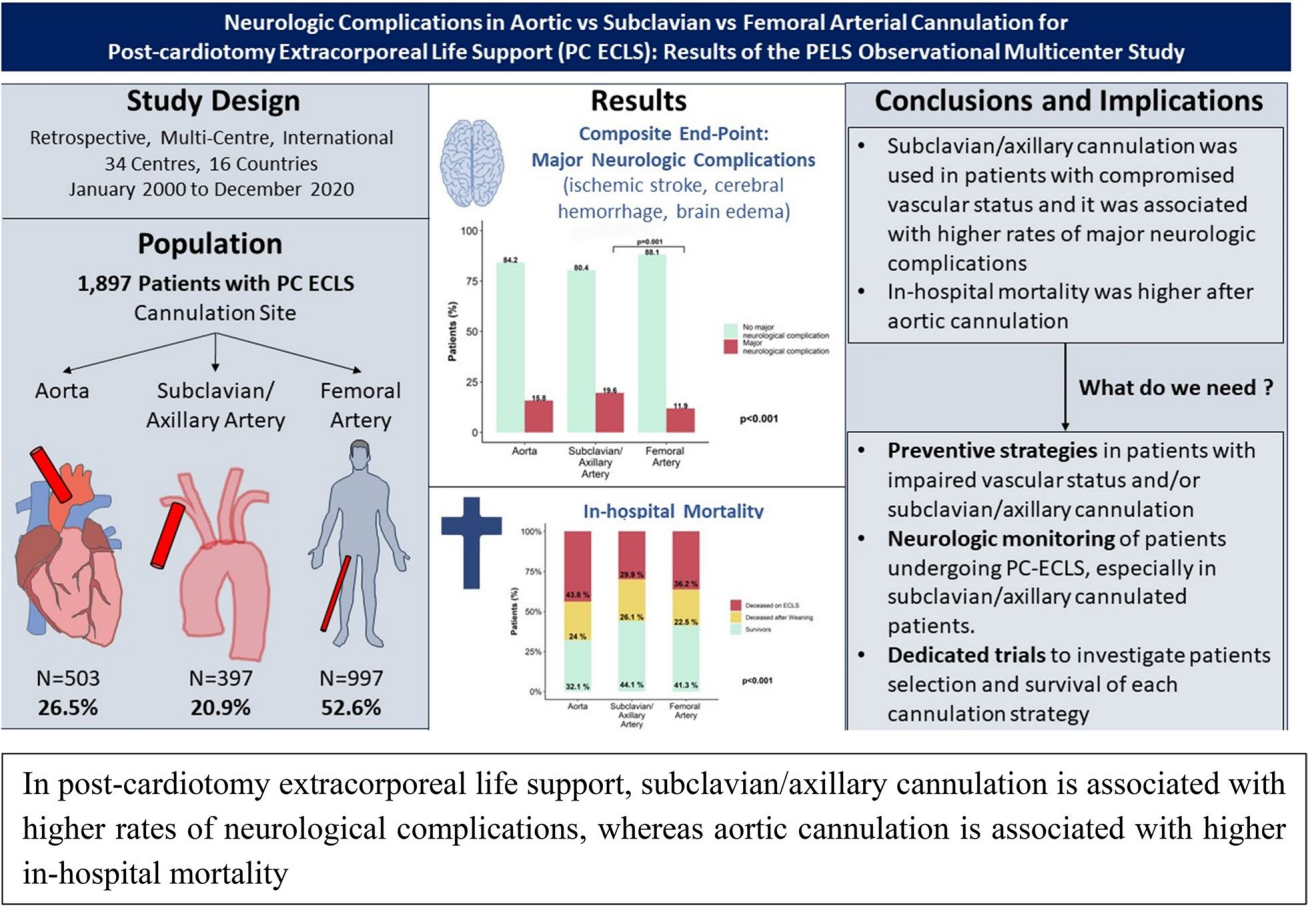

**Supplementary Information:**

The online version contains supplementary material available at 10.1186/s13054-024-05047-2.

## Background

Extracorporeal life support (ECLS) represents a strategy of temporary mechanical circulatory support for refractory post-cardiotomy (PC) cardiogenic shock [1, 2]. ECLS has gained an important role in perioperative care, especially in patients with high-risk profiles undergoing complex cardiac surgical procedures [3]. Nonetheless, morbidity and mortality in such patients are consistently high, with minimal evidence available on long-term and functional outcomes [3, 4]. Particularly, data regarding patients’ neurological outcomes and association with different cannulation approaches in PC-ECLS are lacking and urgently needed to understand the pathophysiology of these complications, monitor and prevent them [5–7]. Since cerebral perfusion patterns during ECLS may be different depending on the arterial cannulation site (antegrade vs. retrograde flow in ascending or descending aorta), the baseline vascular condition or hypoxia, the choice of the return site could potentially lead to a different likelihood of neurologic complications. Nevertheless, evidence on this topic is scarce [8] and the assessment of cerebral autoregulation in relation to cannulation settings or ECLS patient’s characteristics is complex. Moreover, little evidence addressing the relationship between the return cannulation site and neurologic injury exists to inform our cannulation strategy in PC-ECLS patients. In this study, we hypothesize that different ECLS cannulation strategies are associated with different rates of major neurological outcomes including stroke, cerebral bleeding and brain edema. We aim to compare patients' neurologic outcomes in three commonly used ECLS arterial cannulation sites: aortic vs subclavian/axillary vs femoral artery.

## Methods

The multicenter, retrospective observational Post-cardiotomy Extracorporeal Life Support (PELS) study enrolled consecutive patients supported with ECLS in the post-operative phase (ClinicalTrials.gov: NCT03857217) in 34 centers from 16 countries. Adults (≥ 18 years old) were included if they underwent veno-arterial (V-A) ECLS implantation during or after cardiac surgery between January 2000 and December 2020. For the present analysis, we compared three cannulation strategies analyzing preoperative, intra-operative and post-operatory characteristics, neurologic complications and in-hospital mortality (Supplementary Table 1 and 2). Exclusion criteria were veno-venous configuration, mixed cannulations, pulmonary artery cannulation, and cannulation sites unknown (Supplementary Fig. 1).

The current study was conducted in accordance with the Declaration of Helsinki. Institutional Review Board (IRB) approval was acquired in all centers based on the IRB-approval of the coordinating center (METC-2018–0788). The need for informed consent was waived due to the observational character of the study, the emergency of the performed procedure, and the de-identification of shared data. Study was performed following the Strengthening the Reporting of Observational Studies in Epidemiology (STROBE) statement (*Appendix*) [9].

### Data collection and outcomes

Data were collected centrally according to data-sharing agreements between participating centers [3]. Demographics, pre-operative clinical, procedural characteristics, ECLS details, in-hospital morbidity and mortality, and post-discharge survival were included in a dedicated form (data.castoredc.com). Follow-up data were collected through the review of the most recent medical records or contact with patients at discretion of the treating center. The primary outcome was a composite end-point of major neurologic complications (ischemic stroke, cerebral hemorrhage, brain edema). Secondary outcomes included seizures, all-cause in-hospital mortality, neurologic complication as cause of in-hospital mortality and mortality at follow-up after hospital discharge.

### Statistical analysis

The full cohort was categorized into three study groups based on arterial cannulation site (ascending aorta, subclavian/axillary artery, femoral artery). Missing data analysis (Supplementary Table 2) was conducted with the mice: Multivariate Imputation by Chained Equations (MICE) R package [10]. Missing data patterns were investigated and were identified as missing completely at random (MCAR). Descriptive statistics were conducted on available data only and no imputations were performed for this purpose. Normality was tested for continuous variables. Continuous variables were reported as median and interquartile range (IQR) and analyzed with Mann Whitney U-test. Chi-square Test and Fisher’s Exact Test were used to compare group differences for categorical variables expressed as count (percentage). In case of significant differences between groups, post-hoc comparisons were performed and adjusted by the Bonferroni correction for multiple tests (Supplementary Table 3). A *p*-value < 0.05 was considered statistically significant. Survival was investigated with the Kaplan–Meier method and comparisons were performed with Log-rank test. Based on the possible variations in ECLS management over the study period and the confounding factors represented by cardiac arrest, previous stroke, previous transient ischemic attack, peripheral artery disease (PAD), two sensitivity analysis were performed after exclusion of patients who received post-cardiotomy ECLS before 2010 (2010–2020 cohort) and those with the abovementioned conditions. To estimate the associations between type of cannulation (reference group: femoral cannulation) and composite end-point of major neurological complications, we conducted a mixed-effects multivariable logistic regression, using the lme4: Linear Mixed-Effects Models using 'Eigen' and S4 R package [11]. The random effect was used to account for dependency of observations due to clustering in centers and in years. We first estimated a crude model, which was subsequently adjusted for sets of variables deemed potential confounders for the association with the outcome: Model 1, crude model with variable “Arterial cannulation site”; Model 2, arterial cannulation site, PAD; Model 3, arterial cannulation site, PAD, stroke; Model 4, arterial cannulation site, PAD, stroke, interaction stroke*PAD; Model 5, arterial cannulation site, PAD, stroke, hypertension, dialysis, diabetes, preoperative cardiac arrest; Model 6, arterial cannulation site, PAD, stroke, hypertension, dialysis, diabetes, preoperative cardiac arrest, emergency surgery, coronary artery bypass graft (CABG), aortic surgery, cardiopulmonary bypass (CPB) time; Model 7, arterial cannulation site, PAD, stroke, hypertension, dialysis, diabetes, preoperative cardiac arrest, emergency surgery, CABG, aortic surgery, CPB time, implant timing, cardiac arrest as indication for ECLS. Data were merged and analyzed using SPSS 26.0 (IBM, New York, USA), and R 4.4.0 (R Foundation for Statistical Computing, Vienna, Austria).

## Results

### Baseline, surgical, and ECLS characteristics

Overall, 1897 PC-ECLS patients were included in the current study: 503 patients (26.5%) underwent aortic, 397 (20.9%) subclavian/axillary and 997 (52.6%) femoral cannulation (Table 1). Each center enrolled a median value of 24 patients per center (Supplementary Fig. 2). A history of hypertension (*p* < 0.001), smoking (*p* = 0.013), diabetes (*p* = 0.002), previous stroke (p = 0.007), previous myocardial infarction (*p* = 0.002), and dialysis (*p* = 0.041) was more frequent in the subclavian/axillary cannulation group (Table 1 and Supplementary Table 3). PAD was less frequent in femoral cannulation (n = 99, 9.9%), compared to aortic (n = 96, 19.1%) and subclavian/axillary cannulation (n = 86, 21.7%; *p* < 0.001; Table 1 and Supplementary Table 3). A higher rate of pre-operative cardiac arrest was observed in subclavian/axillary cannulation (*p *= 0.005). Emergency surgery occurred less frequently before femoral cannulation (p < 0.001) and CABG was more frequent in subclavian/axillary and aortic than femoral cannulation (*p* < 0.001, Table 2). Cardiopulmonary bypass (CPB) time was longer in aortic (215 min [IQR 143–294 min]) and subclavian/axillary (218 min [IQR 161–307 min]) groups, compared to femoral cannulation (192 min [IQR 129–272 min], *p* < 0.001, Table 2 and Supplementary Table 3).

**Table 1:** Pre-operative Characteristics of the Overall Population

	Aorta (n = 503)		Subclavian/Axillary Artery (n = 397)		Femoral Artery (n = 997)		*P*-value
Age (years)	65	(55–72)	67	(57–74)	64	(55–71)	< 0.001
Sex							
Female	197	(39.2%)	187	(47.1%)	391	(39.3%)	0.018
Male	306	(60.8%)	210	(52.9%)	605	(60.7%)	
Body surface area (m^2^)	1.90	(1.73–2.04)	1.93	(1.79–2.07)	1.87	(1.73–2.023)	0.001
Comorbidities							
Hypertension	335	(66.6%)	298	(75.1%)	590	(63.1%)	< 0.001
Dialysis	41	(8.5%)	44	(12.1%)	76	(7.7%)	0.041
Previous myocardial infarction	146	(29.0%)	129	(32.5%)	237	(23.8%)	0.002
Previous endocarditis	35	(7.0%)	46	(11.6%)	70	(7.0%)	0.011
Smoking	116	(24.0%)	105	(36.0%)	211	(25.3%)	< 0.001
Previous stroke	47	(9.3%)	62	(15.6%)	145	(14.5%)	0.007
Atrial fibrillation	129	(25.6%)	125	(31.6%)	251	(25.2%)	0.044
Previous pulmonary embolism	10	(2.0%)	11	(3.8%)	11	(1.2%)	0.016
Diabetes mellitus	144	(28.6%)	115	(29.0%)	228	(22.9%)	0.013
Previous transient ischemic attack	10	(2.1%)	6	(1.7%)	20	(2.4%)	0.718
Chronic obstructive pulmonary disease	36	(7.2%)	50	(15.2%)	104	(10.5%)	0.001
Peripheral artery disease	96	(19.1%)	86	(21.7%)	99	(9.9%)	< 0.001
Left ventricular ejection fraction (%)	47.0	(30–55)	40.0	(25–60)	48.00	(30–60)	0.002
Euroscore ii	8.0	(3.2–18.1)	14.50	(6.4–29.6)	5.94	(2.4–16.0)	< 0.001
Preoperative conditions							
Nyha class							
Class I	32	(6.8%)	29	(7.5%)	73	(7.6%)	< 0.001
Class II	98	(20.9%)	64	(16.5%)	228	(23.7%)	
Class III	185	(39.5%)	128	(32.9%)	401	(41.7%)	
Class IV	153	(32.7%)	168	(43.2%)	260	(27.0%)	
Preoperative cardiogenic shock	128	(25.4%)	95	(25.5%)	177	(17.8%)	< 0.001
Preoperative cardiac arrest	50	(10.1%)	49	(12.7%)	72	(7.3%)	0.005
Preoperative right ventricular failure	49	(10.1%)	41	(13.5%)	82	(9.4%)	0.125
Preoperative biventricular failure	33	(7.1%)	30	(11.8%)	50	(6.4%)	0.018
Emergency surgery	162	(32.2%)	122	(32.4%)	208	(20.9%)	< 0.001

Data are reported as n (% as valid percentage excluding missing values) or median (interquartile range). NYHA, New York Heart Association

**Table 2:** Procedural Characteristics

	Aorta (N = 503)		Subclavian/Axillary Artery(n = 397)		Femoral Artery(N = 997)		*P*-value
Weight of surgery							
Unknown	4	(0.8%)	0	(0.0%)	7	(0.7%)	< 0.001
Isolated coronary artery bypass graft	93	(18.5%)	62	(15.6%)	177	(17.8%)	
Isolated non coronary artery bypass graft	302	(60.0%)	159	(40.1%)	599	(60.1%)	
2 procedures	16	(3.2%)	64	(16.1%)	59	(5.9%)	
3 or more procedures	88	(17.5%)	112	(28.2%)	155	(15.5%)	
Coronary artery bypass graft	238	(47.3%)	202	(50.9%)	399	(40.0%)	< 0.001
Aortic valve surgery	171	(34.0%)	157	(39.5%)	338	(33.9%)	0.114
Aortic valve surgery type							
Aortic valve repair	23	(17.2%)	41	(35.7%)	49	(20.3%)	0.006
Biological prosthesis	79	(59.0%)	55	(47.8%)	134	(55.6%)	
Mechanical prosthesis	32	(23.9%)	19	(16.5%)	58	(24.1%)	
Mitral valve surgery	158	(31.4%)	143	(36.0%)	306	(30.7%)	0.152
Mitral valve surgery type							
Mitral valve repair	44	(38.6%)	53	(46.5%)	95	(41.7%)	0.537
Biological prosthesis	42	(36.8%)	38	(33.3%)	71	(31.1%)	
Mechanical prosthesis	28	(24.6%)	23	(20.2%)	62	(13.0%)	
Tricuspid valve surgery	63	(12.5%)	66	(16.6%)	126	(12.6%)	0.112
Aortic surgery	98	(19.5%)	84	(21.2%)	182	(18.3%)	0.453
Aortic surgery type							
Aortic root	13	(13.3%)	13	(15.9%)	27	(15.0%)	0.170
Ascending aorta and root	30	(30.6%)	23	(28.0%)	54	(30.0%)	
Ascending aorta	27	(27.6%)	27	(32.9%)	41	(22.8%)	
Ascending aorta and arch	27	(27.6%)	16	(19.5%)	42	(23.3%)	
Aortic arch and descending aorta	1	(1.0%)	3	(3.7%)	16	(8.9%)	
Left ventricular assist device	6	(1.2%)	5	(1.3%)	11	(1.1%)	0.967
Right ventricular assist device	5	(1.0%)	1	(0.3%)	0	(0.0%)	0.005
Heart transplantation	38	(7.6%)	44	(11.1%)	102	(10.2%)	0.147
Off-pump surgery	15	(3.0%)	7	(1.8%)	50	(5.1%)	0.009
Conversion to cardiopulmonary bypass	6	(40.0%)	4	(57.1%)	11	(20.8%)	0.067
Cardiopulmonary bypass time (min)	215	(143–294)	218	(161–307)	192	(129–272)	< 0.001
Crossclamp time (min)	102	(65–152)	102	(68–154)	97	(62–146)	0.196
Intraoperative transfusions	209	(90.1%)	50	(100.0%)	437	(91.4%)	0.071

Data are reported as n (% as valid percentage excluding missing values) or median (interquartile range)

Most common indication for ECLS was failure to wean from cardiopulmonary bypass which was more common in the subclavian/axillary group compared to others (*p* < 0.001) (Table 3). Active left ventricular unloading strategies were applied in 205 patients (49.6%) in aortic, 43 patients (15.0%) in subclavian/axillary and 237 patients (27.4%) in femoral cannulation (*p* < 0.001). Patients were supported on ECLS for a median of 120 h (IQR, 56–206 h) in aortic, 116 h (IQR, 69–182 h) in subclavian/axillary and 116 h (IQR, 52–192 h) in femoral group (p = 0.557). Number of units of post-operatively transfused erythrocyte concentrates was similar in all groups (Supplementary Table 4).

**Table 3:** Details on extracorporeal life support

	Aorta (n = 503)		Subclavian/Axillary Artery (n = 397)		Femoral Artery (N = 997)		*P*-value
ECLS indication							
Failure to wean	204	(40.9%)	195	(52.0%)	347	(35.0%)	< 0.001
Acute pulmonary embolism	0	(0.0%)	0	(0.0%)	2	(0.2%)	
Arrhythmia	12	(2.4%)	4	(1.1%)	26	(2.6%)	
Cardiac arrest	47	(9.4%)	12	(3.2%)	95	(9.6%)	
Cardiogenic shock	127	(25.5%)	71	(18.9%)	281	(28.4%)	
Pulmonary hemorrhage	3	(0.6%)	1	(0.3%)	3	(0.3%)	
Right ventricular failure	58	(11.6%)	41	(10.9%)	114	(11.5%)	
Respiratory failure	7	(1.4%)	24	(6.4%)	24	(2.4%)	
Biventricular failure	34	(6.8%)	23	(6.1%)	82	(8.3%)	
Other	7	(1.4%)	4	(1.1%)	17	(1.7%)	
Left ventricular unloading	205	(49.6%)	43	(15.0%)	237	(27.4%)	< 0.001
ECLS duration (hours)	120	(56–206)	116	(69–182)	116	(52–192)	0.557
Anticoagulation regimen							
None	54	(11.1%)	20	(5.3%)	79	(8.2%)	0.010
Heparin	429	(87.9%)	355	(94.4%)	881	(91.0%)	
Bivalirudin	3	(0.6%)	0	(0.0%)	0	(0.0%)	
Argatroban	1	(0.2%)	0	(0.0%)	4	(0.4%)	

Data are reported as n (% as valid percentage excluding missing values) or median (interquartile range). ECLS, Extracorporeal Life Support

### Neurological outcomes and associated variables

**Fig. 1:**
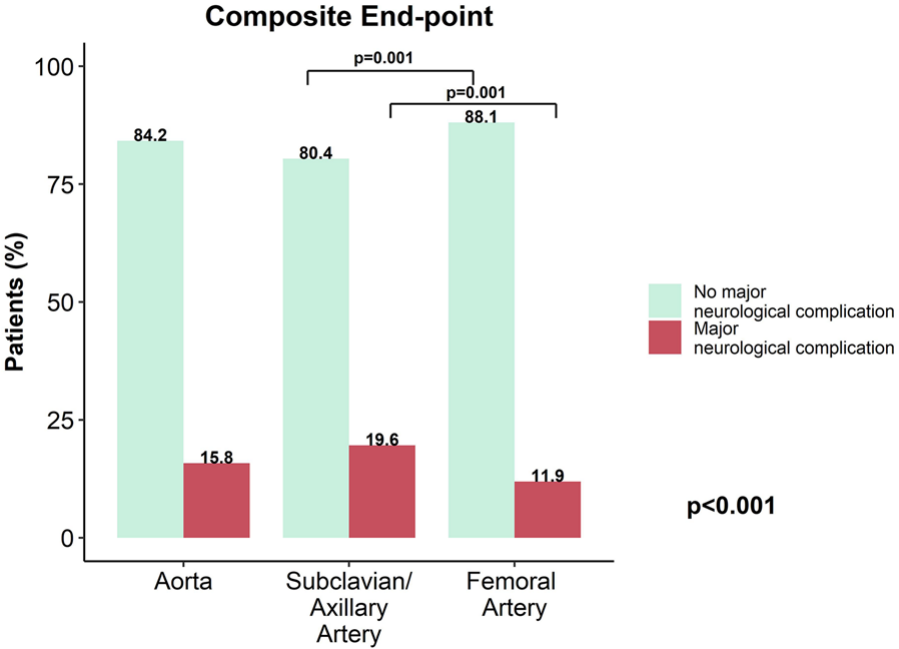
Composite neurologic end-point including stroke, cerebral hemorrhage and brain edema

The composite end-point of major neurological complications was differently distributed between subclavian/axillary (n = 78, 19.6%), aortic (n = 79, 15.8%) and femoral cannulation (n = 118, 11.9%, *p* < 0.001) with subclavian/axillary cannulation showing a higher rate of events compared to femoral cannulation (*p* = 0.001; Fig. 1). Cerebral hemorrhage (aortic: n = 16, 3.2%; subclavian/axillary: n = 22, 5.9%; femoral: n = 21, 2.3%, *p* = 0.004) and stroke (aortic: n = 59, 11.8%; subclavian/axillary: n = 63, 15.9%; femoral: n = 80, 8.1%, *p* < 0.001) were more frequent in patients cannulated with subclavian/axillary approach. Seizures were more common in subclavian/axillary (n = 13, 3.4%) compared to aortic (n = 9, 1.8%) and femoral cannulation (n = 12, 1.3%, p = 0.036). No difference between groups was observed in cerebral hemorrhage severity (*p* = 0.051) and stroke severity (*p* = 0.197, Table 4). Neuro-monitoring was used very infrequently (Table 5). Regression models with random effects for centers and years, subclavian/axillary cannulation was associated with higher risk of neurological complications (OR 1.50 [95% CI 1.05–2.15], *p* = 0.027), also after adjustment for peripheral artery disease, stroke, hypertension, dialysis, diabetes, preoperative cardiac arrest, emergency surgery, CABG, aortic surgery, CPB time, implant timing, cardiac arrest as indication for ECLS (OR 1.53 [95% CI 1.02–2.31], *p* = 0.041; Table 6).

**Table 4:** Post-operative outcome

	Aorta (n = 503)		Subclavian/Axillary Artery(n = 397)		Femoral Artery(n = 997)		*P*-value
Intensive care unit stay (days)	12	(5–25)	16	(8–33)	13	(6–24)	< 0.001
Hospital stay (days)	17	(6–35)	24	(10–46)	20	(8–39)	< 0.001
Postoperative bleeding	339	(68.6%)	217	(54.9%)	526	(53.5%)	< 0.001
Requiring rethoracotomy	234	(47.6%)	135	(43.7%)	347	(35.5%)	< 0.001
Cannulation site bleeding	58	(11.8%)	54	(13.8%)	110	(11.2%)	0.400
Diffuse no-surgical related bleeding	165	(33.5%)	90	(28.3%)	194	(21.2%)	< 0.001
Composite endpoint of neurological outcomes	79	(15.8%)	78	(19.6%)	118	(11.9%)	< 0.001
Brain edema	17	(3.4%)	22	(5.8%)	37	(4.0%)	0.098
Cerebral hemorrhage	16	(3.2%)	22	(5.9%)	21	(2.3%)	0.004
Severity:							
Minor	7	(53.8%)	7	(43.8%)	4	(33.3%)	0.051
Disabling	2	(15.4%)	8	(50.0%)	2	(16.7%)	
Fatal	4	(30.8%)	1	(6.3%)	6	(50.0%)	
Seizure	9	(1.8%)	13	(3.4%)	12	(1.3%)	0.036
Stroke	59	(11.8%)	63	(15.9%)	80	(8.1%)	< 0.001
Severity:							
Minor	23	(45.1%)	28	(58.3%)	26	(38.8%)	0.197
Disabling	18	(35.3%)	14	(29.2%)	22	(32.8%)	
Fatal	10	(19.6%)	6	(12.5%)	19	(28.4%)	
Leg ischemia*	4	4.1%	11	3.8%	123	13.7%	< 0.001
Distal femoral perfusion	NA	(NA)	NA	(NA)	653	74.7%	NA
Arrhythmia	163	(32.6%)	146	(45.5%)	289	(31.3%)	< 0.001
Cardiac arrest	89	(17.8%)	56	(17.5%)	145	(15.7%)	0.540
Bowel ischemia	24	(4.8%)	26	(8.1%)	51	(5.5%)	0.125
Right ventricular failure	106	(21.2%)	95	(33.5%)	167	(18.1%)	< 0.001
Acute kidney injury	272	(54.5%)	225	(70.8%)	483	(52.4%)	< 0.001
Pneumonia	109	(21.8%)	93	(32.7%)	187	(20.3%)	< 0.001
Septic shock	86	(17.2%)	50	(17.7%)	153	(16.6%)	0.901
Distributive shock syndrome	49	(9.8%)	24	(8.5%)	99	(10.7%)	0.536
Acute respiratory distress syndrome	19	(3.8%)	28	(8.7%)	41	(4.4%)	0.003
Multi-organ failure	198	(39.5%)	131	(33.9%)	311	(31.6%)	0.010
Embolism	27	(5.4%)	37	(13.0%)	41	(4.4%)	< 0.001
Main cause of death							
Multiorgan failure	108	(35.2%)	79	(37.3%)	211	(37.8%)	0.433
Sepsis	21	(6.8%)	18	(8.5%)	43	(7.7%)	
Persistent heart failure	124	(40.4%)	77	(36.3%)	197	(35.3%)	
Vasoplegia	7	(2.3%)	2	(0.9%)	11	(2.0%)	
Bleeding	21	(6.8%)	7	(3.3%)	32	(5.7%)	
Neurological injury	12	(3.9%)	14	(6.6%)	28	(5.0%)	
Bowel ischemia	7	(2.3%)	2	(0.9%)	11	(2.0%)	
Other	7	(2.3%)	13	(6.1%)	25	(4.5%)	

Data are reported as n (% as valid percentage excluding missing values) or median (interquartile range); *Data on Leg Ischemia are reported only for Femoral Cannulated patients. NA, Not Available

**Table 5:** Neurological monitoring data

Monitoring tool	Missing data	Aorta (n = 503)	Subclavian/axillary artery(n = 397)	Femoral Artery(n = 997)	*P*-value
Near infrared spectroscopy	491 (25.9%)	94 (20.5%)	8 (4.3%)	178 (23.4%)	< 0.001
Transcranial doppler	490 (25.8%)	2 (0.4%)	0 (0.0%)	3 (0.4%)	0.673
Electroencephalogram	490 (25.8%)	12 (2.6%)	0 (0.0%)	38 (5.0%)	0.002
Brain computed tomography	423 (22.3%)	57 (12.3%)	51 (21.5%)	155 (20.0%)	< 0.001
Brain biomarkers	491 (25.9%)	30 (6.6%)	3 (1.6%)	64 (8.4%)	0.004

Data are reported as n (% as valid percentage excluding missing values)

**Table 6:** The Association between Cannulation and Composite Endpoint of Neurological Outcomes by Mixed-Logistic Regression Analyses. (N = 1560). Reference Group for Cannulation: Femoral Artery

	Aorta (n = 503)		Subclavian/Axillary Artery (n = 397)	
	OR (95% CI)	*P*-value	OR (95% CI)	*P*-value
Model 1. Crude model with variable “Arterial cannulation site”	1.33 (0.97–2.18)	0.079	1.50 (1.05–2.15)	0.027
Model 2. Model 1 + Peripheral artery disease	1.32 (0.96–1.83)	0.088	1.49 (1.04–2.14)	0.029
Model 3. Model 2 + Stroke	1.33 (0.96–1.84)	0.083	1.49 (1.04–2.13)	0.030
Model 4. Model 3 + interaction stroke*Peripheral artery disease	1.33 (0.96–1.84)	0.084	1.50 (1.04–2.14)	0.028
Model 5. Model 3 + hypertension, dialysis, diabetes, preoperative cardiac arrest	1.34 (0.96–1.87)	0.082	1.45 (1.00–2.11)	0.049
Model 6. Model 5 + emergency surgery, coronary artery bypass graft, aortic surgery, cardiopulmonary bypass time	1.32 (0.93–1.87)	0.120	1.42 (0.96–2.11)	0.078
Model 7. Model 6 + Implant timing, Cardiac arrest as indication for ECLS	1.39 (0.98–1.99)	0.067	1.53 (1.02–2.31)	0.041

Data are odds ratios (OR) with 95% confidence intervals (95% CI) for aortic or subclavian/axillary cannulation compared to femoral cannulation (as reference). A higher OR indicates an increased composite end-point of major neurological complications compared to femoral cannulation. CI, Confidence Interval. ECLS, Extracorporeal Life Support

### Secondary outcomes

**Fig. 2:**
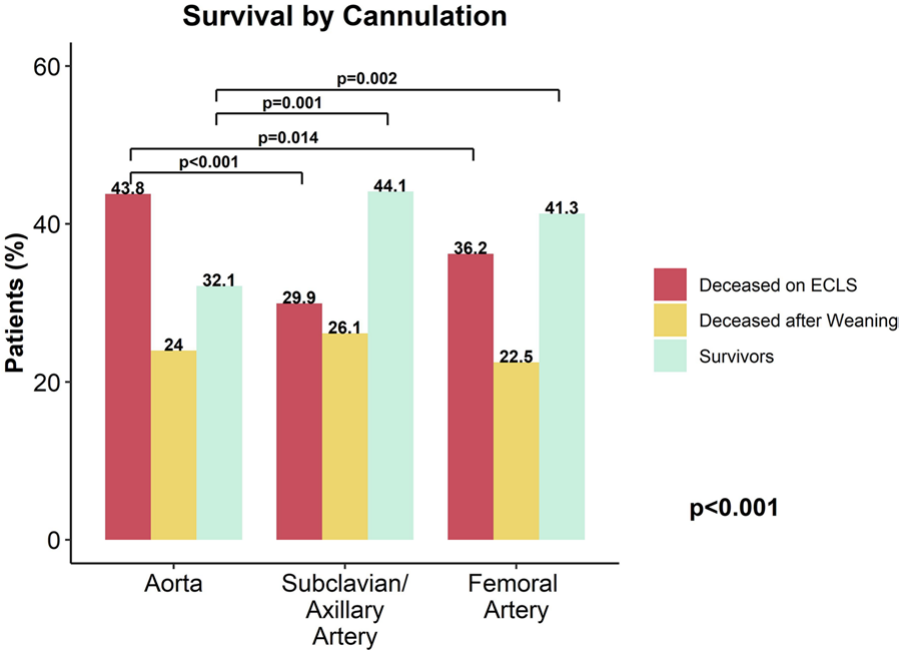
Overall In-hospital Mortality. ECLS = Extracorporeal Life Support

**Fig. 3:**
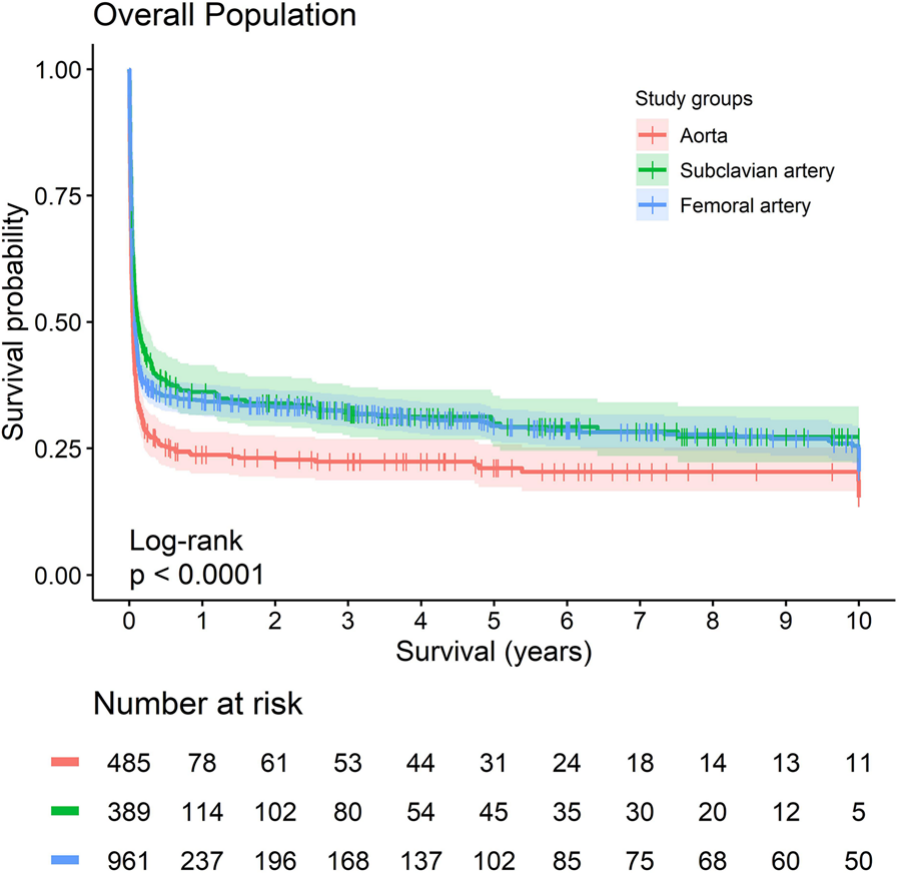
Kaplan–meier plot of survival in subclavian artery versus femoral artery versus aorta cannulation

Intensive care unit stay was longer in subclavian/axillary than aortic or femoral cannulation (*p* < 0.001, Table 4), with a higher in-hospital mortality in the aortic group (aortic: n = 344, 68.4%; subclavian/axillary: n = 223, 56.2%; femoral: n = 587, 58.9%; *p* < 0.001, Fig. 2 and Supplementary Table 3), also confirmed by Kaplan–Meier curves (Fig. 3). More patients deceased during ECLS support in the aortic cannulation group (n = 217, 43.8%) compared to subclavian/axillary (n = 103, 29.9%) and femoral groups (n = 360, 36.3%, *p* < 0.001 Supplementary Table 3). No differences were found in main causes of in-hospital death between groups (*p* = 0.433). Median Follow-up time was 11 days for in-hospital deaths (IQR: 4–22) and 885 days for hospital survivors (IQR: 91–1916).

### Sensitivity analysis

In the sensitivity analysis excluding patients supported before 2010 (Supplementary Tables 5–8), we confirmed the same distribution of the primary outcome and in-hospital mortality observed in the main analysis (Supplementary Fig. 3–5). In the sensitivity analysis excluding patients who suffered previous stroke, previous transient ischemic attack, peripheral vessel disease or with cardiac arrest during hospital stay (Supplementary Tables 9–12), the composite neurological end-point occurred more frequently in the subclavian/axillary group (aortic: n = 48, 15.6%; subclavian/axillary: n = 38, 18.0%; femoral: n = 62, 9.2%; *p* = 0.001; Supplementary Fig. 6). In-hospital mortality remained higher in the aortic cannulation group (aortic: n = 215, 69.8%; subclavian/axillary: n = 103, 48.8%; femoral: n = 370, 56.3%; *p* < 0.001; Supplementary Fig. 7–8).

## Discussion

This study investigated neurological complications in one of the largest cohorts of PC-ECLS patients. The study has four main findings. First, femoral cannulation remains the most used cannulation approach (53%), while subclavian/axillary cannulation (21%) and aortic cannulation (26%) are less used. Second, we found that patients with subclavian/axillary cannulation had worse baseline vascular conditions as shown by higher prevalence of previous stroke, previous myocardial infarction, dialysis, preoperative cardiac arrest and peripheral artery disease. Third, we showed that the composite outcome of neurological complications occurred in almost 20% of patients cannulated with subclavian/axillary, compared to 12–16% of patients cannulated with femoral and aortic approach. Finally, we found higher in-hospital mortality in the aortic group, despite the lower incidence of neurological complications, confirmed by two sensitivity analyses after exclusion of patients supported before 2010 and those with cardiac arrest, stroke, previous transient ischemic attack, peripheral vessel disease.

Overall neurological complications occur in almost 20% of V-A ECLS patients as reported by literature [12]. However, it is still difficult to identify which patient might develop such a complication, its relationship with cerebral autoregulation, its predicted severity, or whether a specific ECLS setting may be related to more neurological events. Moreover, effective strategies to prevent such complications or monitor their early onset are urgently needed to improve PC-ECLS outcomes. Literature supports the hypothesis that cerebral perfusion during ECLS might be influenced by the arterial cannulation site, but whether this could influence neurologic complications is still unknown [13, 14]. Arterial cannulation approach may significantly vary, as demonstrated by this analysis of the PC-ECLS population, with femoral cannulation used in 52.6% of patients, subclavian/axillary cannulation in 20.9% and aortic cannulation used in 26.5% of patients. Femoral cannulation remains the preferred cannulation strategy in PC-ECLS [15], but its superiority in terms of fewer brain embolization events compared to the central aortic one is still debated, as mechanisms underlying the potential beneficial effects of peripheral cannulation have to be confirmed [16–19].

The first step to understanding the association of neurological outcomes and cannulation strategy implies an analysis of patients’ characteristics to identify specific risk profiles for adverse events, identify best candidates for each cannulation strategy, and also understand how clinicians currently select patients for each cannulation approach. This study showed that patients who were cannulated with subclavian/axillary approach were characterized by a worse vascular status compared to others. Moreover, a significant amount of them received emergency surgery and experienced cardiogenic shock or cardiac arrest before the operation. This observation suggests that the subclavian/axillary cannulation is preferred in case of worse patient’s vascular status, possibly because the subclavian/axillary artery is more often free from atheromatic disease compared to femoral vessels or ascending aorta [20, 21]. Surprisingly, subclavian/axillary cannulation was not predominantly used in aortic surgery, even though the subclavian/axillary artery is often cannulated to initiate CPB during this type of operation [20]. Aortic surgery is usually associated with higher neurological risks due to the possible disease involvement of neck vessels or the need for a circulatory arrest [16, 22], but interaction between disease extension and ECLS cannulation approach needs further investigations.

CPB time was slightly longer in patients cannulated with subclavian/axillary and aortic approach compared to femoral approach. This could be explained by the higher surgical complexity that characterized the subclavian/axillary group, whose patients underwent two or more procedures in 28% of cases compared to the 16–18% of other groups. A longer CPB time might influence cerebral circulation and neurological outcomes. However, the interaction between CPB time and flow, intra-operative CPB cannulation and ECLS cannulation sites, hemodynamic parameters, and patient’s vascular status is still under-investigated and requires the development of dedicated studies.

Observations from this study demonstrated that the composite neurological end-point including stroke, brain hemorrhage and brain oedema occurs more frequently in subclavian/axillary cannulation, with 6% of patients suffering cerebral hemorrhage and 16% of patients suffering ischemic stroke in the subclavian/axillary group. Also, clinical seizures were more common in subclavian/axillary patients. The adjusted analysis, which accounted for a range of the ECLS-independent confounding factors, demonstrated that the subclavian/axillary cannulation site remained independently associated with increased neurologic complications. This finding underscores the significance of the cannulation strategy in influencing patient outcomes during ECLS and reduces the likelihood that the observed differences were merely due to the subclavian/axillary group being sicker already before the cannulation. Although tracing the pathophysiological mechanism of these events was beyond the scope of this observational study, we can hypothesize an additive effect of baseline cerebral and vascular comorbidities, an intra-operative alteration of the cerebral blood flow, the lack of pulsatility due to ECLS, an altered cerebral autoregulation and the flow pattern determined by the arterial cannulation site. Moreover, an autoregulatory dysfunction may contribute to neurological dysfunction, but how it interacts with ECLS and cannula flow is still unknown [13, 14, 23, 24] and mechanisms of cerebral autoregulation in the specific setting of non-pulsatile blood flow and after different cannulation strategies are still an active research area [8, 19, 21].

To further investigate all these aspects and allow for an early detection of neurological complications, dedicated neurological monitoring strategies during ECLS are advised even if no specific guidelines still exist. PELS study demonstrated that neurologic monitoring (such as near-infrared spectroscopy, transcranial doppler, electroencephalogram and brain computed tomography) is still not routinely used. However, literature suggests that standardized neuromonitoring, when implemented, significantly improves the detection [25, 26] of acute brain injuries in ECLS patients; particularly lack of blood flow monitoring may be associated with increased stroke incidence [27–29]. Strong heterogeneity exists also in anticoagulation protocols and unloading strategies that might play an important role in determining thrombotic and hemorrhagic brain events and cardiac recovery [30–32]. This study demonstrated a less frequent use of left ventricular unloading strategies in the subclavian/axillary group. Considering the different flow pattern with antegrade flow in the descending aorta, subclavian/axillary cannulation is deemed to be less burdened by left ventricular afterload increase and pulmonary edema. This different hemodynamic pattern might induce a less frequent use of left ventricular unloading strategies [33]. Furthermore, institutional practices and protocols for ECLS management can vary significantly, and some centers may have a “lower threshold” for implementing unloading in patients with femoral or aortic cannulation compared to those with subclavian/axillary approach.

Several studies report in-hospital mortality of patients undergoing PC V-A ECLS around 60% with different variations, depending on weight of surgery, patient’s age and center’s expertise [3, 21, 27, 29, 34–36]. Our survival outcomes were also comparable to those presented in the previous studies and reports from the Extracorporeal Life Support Organization (ELSO) registry [21, 37], although variations may be related to the specific characteristics of our patient cohort. This comparison underscores the validity of our results and highlights the importance of considering patient-specific factors when evaluating ECLS outcomes. Our study also confirmed the importance of describing the death timing when reporting on ECLS mortality [38]. For example, we noticed a higher percentage of on-support mortality within the aortic group that significantly contributes the overall higher mortality in this group. This suggests that the inability to wean from ECLS is a critical factor contributing to the increased mortality in the aortic group, likely reflecting a more severe underlying condition or a less favorable response to ECLS support.

This finding opens the discussion on the fate of ECLS patients with neurological damage. Previous studies showed that patients on V-A ECLS support experiencing neurological complications had an increased in-hospital mortality, by a factor of 2–3 [13, 39–41]. Moreover, severe neurological damage could be a reason to withdraw the support. However, neurological complications remain limited in the ECLS population, and they seem not to be the main driver of mortality [39]. Therefore, these complications, especially the "minor" ones, should not push toward support withdrawal.

The observation that neurologic complications were not the primary cause of mortality in our cohort may be partly explained by the extended period required for neurologic recovery. Clinical teams may be inclined to continue ECLS support while awaiting potential neurologic improvement, as early withdrawal could preclude recovery that may take days to weeks to become evident. This approach emphasizes the importance of patient-specific management and multimodal neurologic monitoring to guide decision-making during ECLS support.

As clinical experience accumulates and ECLS becomes more widely used, focused research on neurological monitoring and management of neurological complications are imperative to improve early and long-term outcomes. Particularly, it is warranted to evaluate if improving neurological monitoring, anticoagulation protocols, neurological targeted therapy, prognostication, and follow-up, may mitigate incidence and severity of neurological complications in all patients, and especially in those with bad vascular status and/or subclavian cannulation. Since we included patients from different centers and countries, our results may be applicable to a large variety of patients treated with PC-ECLS.

### Strengths and limitations

PELS is observational by nature, so causal inference is not possible, and it was not designed to specifically investigate vascular diseases (i.e. carotid stenosis) and neurological outcomes with dedicated tools [12, 16, 41]. Details about CPB cannulation strategies during the index operation or timing (on ECLS, before or after ECLS) of neurological complications were not available, preventing any deeper causal investigation. Moreover, specific data on ECLS selection criteria, protocols, anticoagulation and weaning strategies, cannulation technique and personnel (surgical vs percutaneous) are not captured by the database and could therefore not be included in this study. Similarly, intraoperative and postoperative hemodynamic parameters, oxygen delivery and hypoxia, cerebral autoregulation influencing factors, coagulation parameters, anesthesia management protocols, vasopressors and inotropes usage, reasons for withdrawal of ECLS support, post-discharge quality of life, functional status, re-hospitalization events after discharge and follow-up specific data. As previously mentioned, sicker patients with compromised vascular status more frequently received subclavian/axillary. Therefore, we cannot rule out the effects of confounding by severity and indication which is the main limiting factor of this analysis. While we used models to adjust for these confounding variables, we recognize that these adjustments cannot fully eliminate all biases. Since the observational nature of PELS study, we also cannot infer on relations between other complications (acute kidney injury, pneumonia, acute respiratory distress syndrome, embolism, and arrhythmia, etc.) more common after subclavian/axillary, and neurological complications. Furthermore, we had no access to long-term functional status of these patients, therefore additional studies are warranted in this respect. Participation to the PELS study was on a voluntary basis and centers received no funding for this study. Thus, we cannot exclude that some centers did not provide all available data or included all consecutive patients due to lack of resources, despite the actions taken to support a comprehensive and granular data collection (Supplementary Fig. 2). Indeed, we encountered some missing data, especially regarding severity of stroke or cerebral hemorrhage and vasospasm (Supplementary Table 2). Caution should be applied in the interpretation of data regarding post-operative transfusions due to a high percentage of missing data (n = 918/1897, 48.4%), especially in the subclavian/axillary group. Nevertheless, external validity of our study is supported by the large cohort, the use of linear mixed-effects models including center and year as random effects, and the international participation.

## Conclusions

In this cohort of the PC-ECLS Study, subclavian/axillary cannulation was used in patients with compromised vascular status, and it was associated with higher rates of major neurologic complications and seizure, especially compared to femoral cannulation. In-hospital mortality was higher after aortic cannulation, despite no significant differences in incidence of neurological cause of death in these patients. These results focus attention on the application of preventive strategies in patients with impaired vascular status and/or subclavian/axillary cannulation, encouraging dedicated prospective trials. Eventually, this study suggests the need for an adequate neurologic monitoring of patients undergoing PC-ECLS, especially in subclavian/axillary cannulated patients.

### Supplementary Information

Additional file 1.

## Data Availability

No datasets were generated or analysed during the current study.

## Abbreviations

CABG: Coronary artery bypass surgery
CPB: Cardiopulmonary bypass
ECLS: Extracorporeal life support
IQR: Interquartile range
IRB: Institutional review board
NYHA: New York Heart Association
PAD: Peripheral artery disease
PC-ECLS: Post-cardiotomy extracorporeal life support
PC: Post-cardiotomy
V-A: Veno-arterial
