# Supplementary material for: Neurologic complications in patients receiving aortic versus subclavian versus femoral arterial cannulation for post-cardiotomy extracorporeal life support: results of the PELS observational multicenter study
Source: Crit Care. 2024 Aug 7;28:265. doi: 10.1186/s13054-024-05047-2 (PMC11304572; doi:10.1186/s13054-024-05047-2)
Supplement: Supplementary file 1 — Additional file 1. [file 13054_2024_5047_MOESM1_ESM.docx]

**Neurologic Complications in Patients Receiving Aortic vs Subclavian vs Femoral Arterial Cannulation for Post-cardiotomy Extracorporeal Life Support: Results of the PELS Observational Multicenter Study**

**Appendix**

**Corresponding author:**Giovanni Chiarini, M.D.

Cardio-Thoracic Surgery Department

Maastricht University Medical Centre

P. Debyelaan, 25-6202AZ Maastricht-The Netherlands

Email: giovanni.chiarini88@gmail.com

Phone: 00393473926790

**List of PELS Investigators**

Giovanni Chiarini^1^, Silvia Mariani^1,2^, Bas C.T. van Bussel^4^, Samuel Heuts^1^, Michele Di Mauro^1^, Dominik Wiedemann^5^, Anne-Kristin Schaefer^5^, Luca Conci^5^, Diyar Saeed^6^, Jawad Khalil^6^, Sven Lehmann^6^, Matteo Pozzi^7^, Jean-Francois Obadia^7^, Antonio Loforte^8,9^, Luca Botta^8^, Davide Pacini^8^, Udo Boeken^10^, Nikolaos Kalampokas^10^, Robertas Samalavicius^11^, Agne Jankuviene^11^, Karl Bounader^12^, Erwan Flecher^12^, Xiaotong Hou^13^, Jeroen J.H. Bunge^14,15^, Dinis Dos Reis Miranda^15^, Hergen Buscher^16^, Kogulan Sriranjan^16^, Leonardo Salazar^17^, Bart Meyns^18^, Daniel Herr^19^, Michael A Mazzeffi^19^, Sacha Matteucc^20^, Marco Di Eusanio^20^, Sandro Sponga^21^, Igor Vendramin^21^, Graeme MacLaren^22^, Vitaly Sorokin^22^, Kollengode Ramanathan^22^, Claudio Russo^23^, Alessandro Costetti^23^, Francesco Formica^2,24^, Pranya Sakiyalak^25^, Antonio Fiore^26^, Daniele Camboni^27^, Chistof Schmid^27^, Giuseppe Maria Raffa^28^, Rodrigo Diaz^29^, Roberto Castillo^29^, I-wen Wang^30^, Jae-Seung Jung^31^, Jan Belohlavek^32^, Tomas Grus^33^, Vin Pellegrino^34^, Giacomo Bianchi^35^, Marco Solinas^35^, Matteo Pettinari^36^, Alessandro Barbone^37^, José P. Garcia^38^, Kiran Shekar^39^, Glenn Whitman^40^, Roberto Lorusso^1^.

1. Cardio-Thoracic Surgery Department, and Cardiovascular Research Institute Maastricht, Maastricht, The Netherlands.
2. Cardiac Surgery Unit, San Gerardo Hospital, Monza, Italy
3. Universidade de Pernambuco, Pernambuco, Brazil.
4. Department of Intensive Care Medicine, and Cardiovascular Research Institute Maastricht, Maastricht, The Netherlands.
5. Department of Cardiac Surgery, Medical University of Vienna, Vienna, Austria
6. Department of Cardiac Surgery, Leipzig Heart Center, Leipzig, Germany
7. Department of Cardiac Surgery, Louis Pradel Cardiologic Hospital, Lyon, France
8. Division of Cardiac Surgery, IRCCS Azienda Ospedaliero-Universitaria di Bologna, Bologna, Italy.
9. University of Turin, Turin, Italy
10. Department of Cardiac Surgery, Medical Faculty, Heinrich Heine University, Duesseldorf, Germany.
11. II Department of Anesthesiology, Centre of Anesthesia, Intensive Care and Pain management, Vilnius University Hospital Santariskiu Klinikos, Vilnius, Lithuania.
12. Division of Cardiothoracic and Vascular Surgery. Pontchaillou University Hospital, Rennes, France.
13. Center for Cardiac Intensive Care, Beijing Institute of Heart, Lung, and Blood Vessels Diseases, Beijing Anzhen Hospital, Capital Medical University, Beijing, China.
14. Department of cardiology, Erasmus MC, Rotterdam, The Netherlands.
15. Department of Intensive Care Adults, Erasmus MC, Rotterdam, The Netherlands.
16. Department of Intensive Care Medicine, Center of Applied Medical Research, St Vincent's Hospital, Darlinghurst, NSW, Australia.
17. Department of Cardiology, Fundación Cardiovascular de Colombia, Bucaramanga, Colombia.
18. Department of Cardiac Surgery, University Hospitals Leuven and Department of Cardiovascular Sciences, University of Leuven, Leuven, Belgium.
19. Departments of Medicine and Surgery, University of Maryland, Baltimore, USA.
20. SOD Cardiochirurgia Ospedali Riuniti 'Umberto I - Lancisi - Salesi' Università Politecnica delle Marche, Ancona, Italy.
21. Division of Cardiac Surgery, Cardiothoracic Department, University Hospital of Udine, Udine, Italy.
22. Cardiothoracic Intensive Care Unit, National University Heart Centre, National University Hospital, Singapore, Singapore.
23. Cardiac Surgery Unit, Cardiac Thoracic and Vascular Department, Niguarda Hospital, Milan, Italy.
24. Department of Medicine and Surgery, University of Parma, Parma, Italy.
25. Division of Cardiovascular and Thoracic Surgery, Department of Surgery, Faculty of Medicine Siriraj Hospital, Mahidol University, Bangkok, Thailand.
26. Department of Cardio-Thoracic Surgery, University Hospital Henri-Mondor, Créteil, Paris, France.
27. Department of Cardiothoracic Surgery, University Medical Center Regensburg, Regensburg, Germany.
28. Department for the Treatment and Study of Cardiothoracic Diseases and Cardiothoracic Transplantation, IRCCS-ISMETT (Istituto Mediterraneo per i Trapianti e Terapie ad Alta Specializzazione), Palermo, Italy.
29. ECMO Unit, Departamento de Anestesia, Clínica Las Condes, Las Condes, Santiago, Chile.
30. Division of Cardiac Surgery, Memorial Healthcare System, Hollywood, FL 33021, USA.
31. Department of Thoracic and Cardiovascular Surgery, Korea University Anam Hospital, Seoul, South Korea.
32. 2nd Department of Internal Medicine, Cardiovascular Medicine General Teaching Hospital and 1st Faculty of Medicine, Charles University in Prague, Prague, Czech Republic.
33. 2nd Department of Cardiovascular Surgery, First Faculty of Medicine, Charles University and General University Hospital in Prague, Prague, Czech Republic.
34. Intensive Care Unit, The Alfred Hospital, Melbourne, VIC, Australia.
35. Ospedale del Cuore Fondazione Toscana "G. Monasterio", Massa, Italy.
36. Department of Cardiovascular Surgery, Ziekenhuis Oost-Limburg, Genk, Belgium.
37. Cardiac Surgery Unit, IRCCS Humanitas Research Hospital – Rozzano (MI) – Italy.
38. IU Health Advanced Heart & Lung Care, Indiana University Methodist Hospital, Indianapolis, IN, USA.
39. Adult Intensive Care Services, The Prince Charles Hospital, Brisbane, Australia.
40. Cardiac Intensive Care Unit, Johns Hopkins Hospital, Baltimore, Maryland, USA.

**STROBE Statement**. Checklist of items that should be included in reports of observational studies

|  | Item No | Recommendation | Page  No |
| --- | --- | --- | --- |
| **Title and abstract** | 1 | (*a*) Indicate the study’s design with a commonly used term in the title or the abstract | 1 |
|  |  | (*b*) Provide in the abstract an informative and balanced summary of what was done and what was found | 4 |
| Introduction | | | |
| Background/rationale | 2 | Explain the scientific background and rationale for the investigation being reported | 7 |
| Objectives | 3 | State specific objectives, including any prespecified hypotheses | 7 |
| Methods | | | |
| Study design | 4 | Present key elements of study design early in the paper | 8 |
| Setting | 5 | Describe the setting, locations, and relevant dates, including periods of recruitment, exposure, follow-up, and data collection | 8 |
| Participants | 6 | (*a*) *Cohort study*—Give the eligibility criteria, and the sources and methods of selection of participants. Describe methods of follow-up  *Case-control study*—Give the eligibility criteria, and the sources and methods of case ascertainment and control selection. Give the rationale for the choice of cases and controls  *Cross-sectional study*—Give the eligibility criteria, and the sources and methods of selection of participants | 8 |
|  |  | (*b*) *Cohort study*—For matched studies, give matching criteria and number of exposed and unexposed  *Case-control study*—For matched studies, give matching criteria and the number of controls per case |  |
| Variables | 7 | Clearly define all outcomes, exposures, predictors, potential confounders, and effect modifiers. Give diagnostic criteria, if applicable | 8 –  Supplementary methods |
| Data sources/ measurement | 8* | For each variable of interest, give sources of data and details of methods of assessment (measurement). Describe comparability of assessment methods if there is more than one group | 8 –  Supplementary methods |
| Bias | 9 | Describe any efforts to address potential sources of bias | 10 |
| Study size | 10 | Explain how the study size was arrived at | n.a. |
| Quantitative variables | 11 | Explain how quantitative variables were handled in the analyses. If applicable, describe which groupings were chosen and why | 8 |
| Statistical methods | 12 | (*a*) Describe all statistical methods, including those used to control for confounding | 9 |
|  |  | (*b*) Describe any methods used to examine subgroups and interactions | 9 |
|  |  | (*c*) Explain how missing data were addressed | 9 |
|  |  | (*d*) *Cohort study*—If applicable, explain how loss to follow-up was addressed  *Case-control study*—If applicable, explain how matching of cases and controls was addressed  *Cross-sectional study*—If applicable, describe analytical methods taking account of sampling strategy | n.a. |
|  |  | (*e*) Describe any sensitivity analyses | 10 |

Continued on next page

| Results | | | |
| --- | --- | --- | --- |
| Participants | 13* | (a) Report numbers of individuals at each stage of study—eg numbers potentially eligible, examined for eligibility, confirmed eligible, included in the study, completing follow-up, and analysed | 10 |
|  |  | (b) Give reasons for non-participation at each stage | 10 |
|  |  | (c) Consider use of a flow diagram | Supplementary figure 1 |
| Descriptive data | 14* | (a) Give characteristics of study participants (eg demographic, clinical, social) and information on exposures and potential confounders | 10 |
|  |  | (b) Indicate number of participants with missing data for each variable of interest | Supplementary table 2 |
|  |  | (c) *Cohort study*—Summarise follow-up time (eg, average and total amount) | 12 |
| Outcome data | 15* | *Cohort study*—Report numbers of outcome events or summary measures over time | *11-12* |
|  |  | *Case-control study—*Report numbers in each exposure category, or summary measures of exposure |  |
|  |  | *Cross-sectional study—*Report numbers of outcome events or summary measures |  |
| Main results | 16 | (*a*) Give unadjusted estimates and, if applicable, confounder-adjusted estimates and their precision (eg, 95% confidence interval). Make clear which confounders were adjusted for and why they were included | 11-12,  Table 6 |
|  |  | (*b*) Report category boundaries when continuous variables were categorized |  |
|  |  | (*c*) If relevant, consider translating estimates of relative risk into absolute risk for a meaningful time period |  |
| Other analyses | 17 | Report other analyses done—eg analyses of subgroups and interactions, and sensitivity analyses | 12-13 |
| Discussion | | | |
| Key results | 18 | Summarise key results with reference to study objectives | 13-14 |
| Limitations | 19 | Discuss limitations of the study, taking into account sources of potential bias or imprecision. Discuss both direction and magnitude of any potential bias | 16 |
| Interpretation | 20 | Give a cautious overall interpretation of results considering objectives, limitations, multiplicity of analyses, results from similar studies, and other relevant evidence | 15 |
| Generalisability | 21 | Discuss the generalisability (external validity) of the study results | 17 |
| Other information | | | |
| Funding | 22 | Give the source of funding and the role of the funders for the present study and, if applicable, for the original study on which the present article is based | 18 |

*Give information separately for cases and controls in case-control studies and, if applicable, for exposed and unexposed groups in cohort and cross-sectional studies.

**Note:** An Explanation and Elaboration article discusses each checklist item and gives methodological background and published examples of transparent reporting. The STROBE checklist is best used in conjunction with this article (freely available on the Web sites of PLoS Medicine at http://www.plosmedicine.org/, Annals of Internal Medicine at http://www.annals.org/, and Epidemiology at http://www.epidem.com/). Information on the STROBE Initiative is available at www.strobe-statement.org.

**Supplementary Tables:**

**Supplementary Table 1** – Variable and Outcomes Definitions.

| **Variable and outcomes definitions.** | |
| --- | --- |
| **Variable** | **Definition** |
| Baseline Characteristics | |
| Hypertension | Systolic blood pressure >140mmHg or diastolic blood pressure >90mmHg, or use of antihypertensive agents to maintain normal blood pressure |
| Smoking | Active (smoking during the past 30 days) and more than 100 cigarettes during lifetime |
| Copd | Diagnosis of chronic obstructive pulmonary disease, any GOLD classification |
| Peripheral Arterial Disease | Claudication, carotid occlusion or >50% stenosis, amputation for arterial disease or previous or planned intervention on the abdominal aorta, limb arteries or carotids |
| Pulmonary Hypertension | Systolic pulmonary artery pressure >50mmHg |
| Euroscore II | European System for Cardiac Operative Risk Evaluation II proposing a risk assessment of cardiac surgical procedures which incorporates patient age, sex, diabetic status, pulmonary disease, neurological function, renal function, presence of active endocarditis, pre-operative state, procedural urgency and procedure type |
| NYHA Class | Functional class of dyspnea according to the classification as proposed by the New York Heart Association |
| Preoperative Cardiogenic Shock | Preoperative state with life-threatening hypotension despite rapidly escalating inotropic support, critical organ hypoperfusion, with worsening acidosis and/or lactate levels |
| Preoperative Cardiac Arrest | Preoperative cardiopulmonary resuscitation in the 24 hours prior to surgery |
| Preoperative Right Ventricular Failure | Evidence of right-sided structural and/or functional abnormalities in combination with clinical symptoms and signs of RV failure |
| Emergency Surgery | Surgery before the beginning of the next working day after the decision to operate is made |
| Urgent Surgery | Patients not electively admitted for operation but requiring surgery during the current admission without a possibility to be discharged before undergoing the definite procedure |
| Aortic Vessel Disease | And disease of the ascending aorta, aortic arch or proximal descending aorta warranting surgical correction during the current procedure |
| Aortic Valve Disease | Any aortic valve disease, including (prosthetic) aortic valve stenosis, regurgitation and endocarditis |
| Mitral Valve Disease | Any mitral valve disease, including (prosthetic) mitral valve stenosis, regurgitation and endocarditis |
| Tricuspid Valve Disease | Any tricuspid valve disease, including (prosthetic) tricuspid valve stenosis, regurgitation and endocarditis |
| Pulmonary Valve Disease | Any pulmonary valve disease, including (prosthetic) pulmonary valve stenosis, regurgitation and endocarditis |
| Active Endocarditis | Patients still on antibiotic treatment for endocarditis at the time of surgery |
| Details On ECMO | |
| Failure To Wean | Failure to wean from CPB despite preload optimization and completeness of surgery |
| Arrhythmia | Refractory ventricular arrhythmia with uncontrollable hemodynamic consequences |
| Cardiac Arrest | Abrupt loss of heart function despite acute and simple interventions such as pacing and defibrillation |
| Cardiogenic Shock | State of life-threatening hypotension despite rapidly escalating inotropic support, critical organ hypoperfusion, with worsening acidosis and/or lactate levels |
| Right Ventricular Failure | Evidence of right-sided structural and/or functional abnormalities in combination with clinical symptoms and signs of RV failure |
| Respiratory Failure | Reversible pulmonary disease which cannot anymore be managed by conventional mechanical ventilation, despite optimization of pharmacological interventions with or without prone positioning |
| Biventricular Failure | Biventricular dysfunction accompanied by both signs and symptoms of right-sided and left-sided heart failure |
| Chest Closed | Any cannulation condition in which the sternum is closed irrespective location of cannulas |
| Chest Open | Any cannulation condition in which the sternum is left open irrespective of skin closure |
| Postoperative Outcomes | |
| Stroke | Neurological dysfunction caused by focal brain or retinal ischemia with clinical symptoms lasting less more than 24 hours, with or without permanent disability. Stroke severity as defined by Brott et al (1989) by modified ranking scale based on mRS score. Minor stroke = mRS <2. Major stroke = mRS > or equal to 2^1^ |
| Cerebral Hemorrhage | Neurological dysfunction caused by bleeding into the brain parenchyma^2^ |
| Brain Edema | Swelling of the brain caused by numerous etiologies (vasogenic, osmotic, interstitial, cellular, etc)^3^ |
| Seizures | Sudden bursts of electrical activity in the brain that can cause changes in levels of consciousness and/or movements^4^ |
| Arrhythmia | Any atrial or ventricular arrhythmia lasting more than 30 seconds |
| Leg Ischemia | Clinical signs of lower extremity ischemia requiring intervention (either by vascular surgery or cannula removal) |
| Bowel Ischemia | Intestinal ischemia with elevated lactate levels requiring abdominal surgical intervention |
| Acute Kidney Injury | Postoperative requirement for dialysis while not on dialysis before or duplication of preoperative creatinine levels (and absolute creatinine level >177μmol/L) |
| Pneumonia | Any (suspected) pulmonary infection treated with antibiotics |
| Septic Shock | Sepsis with vasopressor requirement to maintain MAP >65mmHg and serum lactate levels greater than 2mmol/L in the absence of hypovolemia^5^ |
| ARDS, Acute Respiratory Distress Syndrome | Acute Respiratory Distress Syndrome. Acute diffuse inflammatory lung injury requiring invasive mechanical ventilation or extracorporeal membrane oxygenation^6^ |
| Multi-Organ Failure | Hypometabolic state with involvement of more than one organ as established by biochemical and/or radiological analysis |

**Supplementary table 2.** Missing Data in Overall Population

| Variable | Missing Data  N (%) |
| --- | --- |
| Baseline conditions data | |
| Age | 1 (0.1%) |
| Sex | 1 (0.1%) |
| Race | 433 (22.8%) |
| Body Surface Area | 12 (0.6%) |
| Hypertension | 62 (3.3%) |
| Dialysis | 61 (3.2%) |
| Previous Myocardial Infarction | 0 (0.0%) |
| Previous Endocarditis | 0 (0.0%) |
| Smoking | 288 (15.2%) |
| Previous Stroke | 0 (0.0%) |
| Atrial Fibrillation | 1 (0.1%) |
| Previous Acute Pulmonary Embolism | 172 (9.1%) |
| Diabetes Mellitus | 0 (0.0%) |
| Previous Transient Ischemic Attack | 207 (10.9%) |
| Chronic Obstructive Pulmonary Disease | 78 (4.1%) |
| Peripheral Artery Disease | 0 (0.0%) |
| Left Ventricle Ejection Fraction | 70 (3.7%) |
| Euroscore II | 519 (27.4%) |
| New York Heart Association | 78 (4.1%) |
| Preoperative Cardiogenic Shock | 28 (1.5%) |
| Preoperative Cardiac Arrest | 22 (1.2%) |
| Preoperative Right Ventricular Failure | 234 (12.3%) |
| Preoperative Biventricular Failure | 397 (20.9%) |
| Emergency Surgery | 22 (1.2%) |
| Procedural Characteristics Data | |
| Coronary Artery Bypass Graft | 0 (0.0%) |
| Aortic Valve Surgery | 0 (0.0%) |
| Mitral Valve Surgery | 1 (0.1%) |
| Tricuspid Valve Surgery | 0 (0.0%) |
| Aortic Surgery | 0 (0.0%) |
| Aortic Surgery Type | 4 (1.1%) |
| Pulmonary Valve Surgery | 0 (0.0%) |
| Left Ventricular Assist Device | 0 (0.0%) |
| Right Ventricular Assist Device | 0 (0.0%) |
| Heart Transplantation | 0 (0.0%) |
| Off Pump Surgery | 24 (1.3%) |
| Conversion To Cardiopulmonary Bypass | 0 (0.0%) |
| Cardiopulmonary Bypass Time | 92 (5.0%) |
| Crossclamp Time | 103 (5.6%) |
| Intraoperative Transfusions | 1137 (59.9%) |
| Details On Extracorporeal Life Support | |
| ECLS Indication | 32 (1.7%) |
| Cannulation Approach | 0 (0.0%) |
| Left Ventricle Unloading | 333 (17.6%) |
| ECLS Duration (Hours) | 18 (0.9%) |
| Post-Operative Outcomes | |
| Intensive Care Unit Stay Duration (Days) | 58 (3.1%) |
| Hospital Stay Duration | 46 (2.4%) |
| Transfusions | 918 (48.4%) |
| Bleeding | 24 (1.3%) |
| Requiring Rethoracotomy | 118 (6.2%) |
| Cannulation Site Bleeding | 27 (1.4%) |
| Diffuse No-Surgical Related Bleeding | 170 (9.0%) |
| Brain Edema | 94 (5.0%) |
| Cerebral Hemorrhage | 97 (5.1%) |
| Seizure | 94 (5.0%) |
| Stroke | 12 (0.6%) |
| Leg Ischemia | 607 (32.0%)* |
| Cardiac Arrest | 154 (8.1%) |
| Bowel Ischemia | 152 (8.0%) |
| Right Ventricle Failure | 190 (10.0%) |
| Acute Kidney Injury | 158 (8.3%) |
| Pneumonia | 190 (10.0%) |
| Septic Shock | 192 (10.1%) |
| Distributive Shock Syndrome | 193 (10.2%) |
| Acute Respiratory Distress Syndrome | 153 (8.1%) |
| Multiple Organ Failure | 26 (1.4%) |
| Vascular Surgery | 220 (11.6%) |
| In-Hospital Mortality | 0 (0.0%) |
| Neurological Monitoring | |
| Near InfraRed Spectroscopy | 491 (25.9%) |
| TransCranial Doppler | 490 (25.8%) |
| ElectroEncephalogram | 490 (25.8%) |
| Brain Computed Tomography | 423 (22.3%) |
| Brain Biomarkers | 491 (25.9%) |
| Data are reported as n (% of total number of patients). ECLS: extracorporeal life support.*missing data n=65 (6.5%) in femoral patients | |

| **Supplementary Table 3.** Post-Hoc Analyses for Significant Between-Group Differences |
| --- |

| Comparisons of Column Proportions^b^ | | | | |
| --- | --- | --- | --- | --- |
|  | | **Aortic** | **Subclavian/Axillary** | **Femoral** |
|  |  | **(A)** | **(B)** | **(C)** |
| Age | (years) |  | A (0.002) C (0.001) |  |
| Sex | Female |  | C (0.022) |  |
|  | Male |  |  | B (0.022) |
| Body Surface Area | (m^2^) |  | A (0.038) C (0.005) |  |
| Left Ventricle Ejection Fraction | (%) | B (0.004) |  | B (0.000) |
| Euroscore II |  |  | A (0.000) C (0.000) |  |
| Hypertension | No | B (0.017) |  | B (0.000) |
|  | Yes |  | A (0.017) C (0.000) |  |
| Dialysis | No |  |  | B (0.037) |
|  | Yes |  | C (0.037) |  |
| Previous Myocardial Infarction | No |  |  | B (0.003) |
|  | Yes |  | C (0.003) |  |
| Smoking | No | B (0.001) |  | B (0.001) |
|  | Yes |  | A (0.001) C (0.001) |  |
| Previous Stroke | No | B (0.013) C (0.013) |  |  |
|  | Yes |  | A (0.013) | A (0.013) |
| Atrial Fibrillation | No |  |  | B (0.046) |
|  | Yes |  | C (0.046) |  |
| Diabetes Mellitus | No |  |  | A (0.044) |
|  | Yes | C (0.044) |  |  |
| Chronic Obstructive Pulmonary Disease | No | B (0.001) |  |  |
|  | Yes |  | A (0.001) |  |
| Peripheral Artery Disease | No |  |  | A (0.000) B (0.000) |
|  | Yes | C (0.000) | C (0.000) |  |
| Previous Cardiac Surgery | No | B (0.001) |  |  |
|  | Yes |  | A (0.001) |  |
| Emergency Surgery | No |  |  | A (0.000) B (0.000) |
|  | Yes | C (0.000) | C (0.000) |  |
| Preoperative Cardiac Arrest | No |  |  | B (0.004) |
|  | Yes |  | C (0.004) |  |
| Preoperative Biventricular Failure | No |  |  | B (0.017) |
|  | Yes |  | C (0.017) |  |
| Coronary Artery Disease | No |  |  | A (0.019) B (0.003) |
|  | Yes | C (0.019) | C (0.003) |  |
| Coronary Artery Bypass Graft | No |  |  | A (0.021) B (0.001) |
|  | Yes | C (0.021) | C (0.001) |  |
| Cardiopulmonary Bypass Time | (min) | C (0.034) | C (0.001) |  |
| Off-pump surgery | No |  | C( ,016) |  |
|  | Yes |  |  | B( ,016) |
| Weight of surgery | Unknown |  | .a |  |
|  | Isolated CABG |  |  |  |
|  | Isolated non-CABG | B( ,000) |  | B( ,000) |
|  | 2 procedures |  | A( ,000) C( ,000) |  |
|  | 3 or more procedures |  | A( ,000) C( ,000) |  |
| Anticoagulation | None | B( ,008) |  |  |
|  | Heparin |  | A( ,003) |  |
|  | Bivalirudin |  | .a | .a |
|  | Argatroban |  | .a |  |
|  | Protamine |  |  |  |
| ECLS Indication | Failure to Wean |  | A (0.003) C (0.000) |  |
|  | Acute Pulmonary Embolism | .^a^ | .^a^ |  |
|  | Arrhythmia |  |  |  |
|  | Cardiac Arrest | B (0.001) |  | B (0.000) |
|  | Cardiogenic Shock |  |  | B (0.001) |
|  | Pulmonary Hemorrhage |  |  |  |
|  | Right Ventricular Failure |  |  |  |
|  | Respiratory Failure |  | A (0.000) C (0.001) |  |
|  | Biventricular Failure |  |  |  |
|  | Other |  |  |  |
| Left Ventricle Unloading | No |  | A (0.000) C (0.000) | A (0.000) |
|  | Yes | B (0.000) C (0.000) |  | B (0.000) |
| Postoperative Bleeding | No |  | A (0.000) | A (0.000) |
|  | Yes | B (0.000) C (0.000) |  |  |
| Leg Ischemia | No | C( ,001) | C( ,000) |  |
|  | Yes |  |  | A( ,001) B( ,000) |
| Postoperative Embolism | No | B( ,001) |  | B( ,000) |
|  | Yes |  | A( ,001) C( ,000) |  |
| Neurological Composite End-Point | No |  |  | B (0.001) |
|  | Yes |  | C (0.001) |  |
| Cerebral Hemorrhage | No |  |  | B (0.003) |
|  | Yes |  | C (0.003) |  |
| Seizures | No |  |  | B(0.032) |
|  | Yes |  | C(0.032) |  |
| Stroke | No |  |  | B (0.000) |
|  | Yes |  | C (0.000) |  |
| Arrhythmia | No | B (0.001) |  | B (0.000) |
|  | Yes |  | A (0.001) C (0.000) |  |
| Right Ventricle Failure | No | B (0.000) |  | B (0.000) |
|  | Yes |  | A (0.000) C (0.000) |  |
| Acute Kidney Injury | No | B (0.000) |  | B (0.000) |
|  | Yes |  | A (0.000) C (0.000) |  |
| Pneumonia | No | B (0.002) |  | B (0.000) |
|  | Yes |  | A (0.002) C (0.000) |  |
| Acute Respiratory Distress Syndrome | No | B (0.009) |  | B (0.012) |
|  | Yes |  | A (0.009) C (0.012) |  |
| Multiple Organ Failure | No |  |  | A (0.007) |
|  | Yes | C (0.007) |  |  |
| Vascular Surgery | No | C (0.001) |  |  |
|  | Yes |  |  | A (0.001) |
| Monitoring: cerebral NIRS | No |  | A( ,000) C( ,000) |  |
|  | Yes | B( ,000) |  | B( ,000) |
| Monitoring: brain biomarkers | No |  | A( ,027) C( ,003) |  |
|  | Yes | B( ,027) |  | B( ,003) |
| Monitoring: EEG | No | C( ,043) | .a |  |
|  | Yes |  | .a | A( ,043) |
| Monitoring: brain CT | No | B( ,004) C( ,001) |  |  |
|  | Yes |  | A( ,004) | A( ,001) |
|  | Yes |  | A( ,000) C( ,001) |  |
| Intensive Care Unit Stay | (days) |  | A (0.000) C (0.000) |  |
| Hospital Stay | (days) |  | A (0.001) C (0.004) |  |
| In-hospital mortality | No |  | A(0.000) | A(0.001) |
|  | Yes | B(0.000) |  |  |
| Survival Group | Survivors |  | A (0.001) | A (0.002) |
|  | Deceased after Weaning |  |  |  |
|  | Deceased on ECLS | B (0.000) C (0,014) |  |  |
| Results are based on two-sided tests. For each significant pair, the key of the category with the smaller column proportion appears in the category with the larger column proportion.  Significance level for upper case letters (A, B, C): .05 | | | | |
| a. This category is not used in comparisons because its column proportion is equal to zero or one. | | | | |
| b. Tests are adjusted for all pairwise comparisons within a row of each innermost subtable using the Bonferroni correction. | | | | |
| ECLS: extracorporeal life support. | | | | |

| **Supplementary Table 4.** Postoperative Transfusions | | | | | | | | | | |  | |
| --- | --- | --- | --- | --- | --- | --- | --- | --- | --- | --- | --- | --- |
|  | **Aorta  (N=503)** | | | **Subclavian/Axillary Artery (n=397)** | | | | **Femoral Artery (N=997)** | | | | **P-value** |
|  |  |  | **Missing values** |  |  | | **Missing values** |  |  | **Missing values** | |  |
| **Postoperative Transfusions**  (number of packed red blood cells) | 10 | (4-23) | 199 (39.6%) | 10.5 | | (1-22) | 357 (89.9%) | 10 | (4-20) | 362 (36.3%) | | 0.626 |
| Data are reported as n(%) or median (interquartile range). | | | | | | | | | | | | |

**Sensitivity Analysis after Excluding Patients who Received a Post-Cardiotomy Extracorporeal Life Support Before 2010**

**Supplementary Table 5.** Baseline Characteristics of the Population 2010-2020.

|  | **Aortic (n=397)** | | **Subclavian/Axillary (n=353)** | | **Femoral (n=803)** | | **P-value** |
| --- | --- | --- | --- | --- | --- | --- | --- |
| Age (years) | 65 | (55-72) | 68 | (57-74) | 64 | (55-71) | 0.003 |
| Sex |  | | | | | | |
| Female | 151 | (38.0%) | 169 | (47.9%) | 283 | (35.3%) | <0.001 |
| Male | 246 | (62.0%) | 184 | (52.1%) | 519 | (64.7%) |  |
| BSA (m^2^) | 1.89 | (1.73-2.05) | 1.93 | (1.79-2.07) | 1.88 | (1.73-2.03) | 0.006 |
| Comorbidities |  | | | | | | |
| Hypertension | 265 | (66.8%) | 269 | (76.2%) | 479 | (64.6%) | <0.001 |
| Dialysis | 32 | (8.2%) | 43 | (12.8%) | 71 | (7.7%) | 0.017 |
| Previous Myocardial Infarction | 109 | (27.5%) | 108 | (30.6%) | 195 | (24.3%) | 0.073 |
| Previous Endocarditis | 32 | (8.1%) | 44 | (12.5%) | 58 | (7.2%) | 0.013 |
| Smoking | 85 | (21.7%) | 92 | (34.6%) | 176 | (24.6%) | <0.001 |
| Previous Stroke | 36 | (9.1%) | 51 | (14.4%) | 123 | (15.3%) | 0.010 |
| Atrial Fibrillation | 104 | (26.2%) | 111 | (31.5%) | 199 | (24.8%) | 0.056 |
| Previous Pulmonary Embolism | 8 | (2.0%) | 11 | (4.3%) | 6 | (0.8%) | 0.001 |
| Diabetes Mellitus | 108 | (27.2%) | 104 | (29.5%) | 123 | (15.3%) | 0.055 |
| Previous Transient Ischemic Attack | 8 | (2.0%) | 6 | (1.8%) | 19 | (2.6%) | 0.673 |
| COPS | 28 | (7.1%) | 44 | (14.9%) | 74 | (9.2%) | 0.002 |
| Peripheral Artery Disease | 71 | (17.9%) | 71 | (20.1%) | 79 | (9.8%) | <0.001 |
| Left Ventricle Ejection Fraction (%) | 47 | (30-55) | 41 | (25-60) | 50 | (34-60) | 0.002 |
| Euroscore II | 8.3 | (3.3-18.4) | 14.3 | (6.4-29.1) | 5.4 | (2.3-14.7) | <0.001 |
| Preoperative Condition | | | | | | | |
| NYHA Class |  | | | | | | |
| Class I | 26 | (7.0%) | 27 | (7.7%) | 65 | (8.4%) | <0.001 |
| Class II | 77 | (20.8%) | 59 | (16.9%) | 196 | (25.4%) |  |
| Class III | 151 | (40.7%) | 117 | (33.5%) | 325 | (42.0%) |  |
| Class IV | 117 | (31.5%) | 146 | (41.8%) | 187 | (24.2%) |  |
| Preoperative Cardiogenic Shock | 101 | (25.4%) | 87 | (25.9%) | 145 | (18.1%) | 0.002 |
| Preoperative Cardiac Arrest | 36 | (9.1%) | 44 | (12.9%) | 40 | (5.0%) | <0.001 |
| Preoperative Right Ventricular Failure | 43 | (11.1%) | 40 | (14.6%) | 54 | (7.5%) | 0.002 |
| Preoperative Biventricular Failure | 28 | (7.4%) | 30 | (13.0%) | 44 | (6.3%) | 0.004 |
| Emergency Surgery | 124 | (31.2%) | 104 | (31.2%) | 159 | (19.8%) | <0.001 |
| Data are reported as n (% as valid percentage excluding missing values) or median (interquartile range). BSA: Body Surface Area; COPD: Chronic Obstructive Pulmonary Disease; NYHA: New York Heart Association. | | | | | | | |

**Supplementary Table 6.** Procedural Characteristics of the Population 2010-2020

|  | **Aortic (n=397)** | | **Subclavian/Axillary (n=353)** | | **Femoral (n=803)** | | **P-value** |
| --- | --- | --- | --- | --- | --- | --- | --- |
| Coronary Artery Bypass Graft | 181 | (45.6%) | 176 | (49.9%) | 335 | (31.7%) | 0.033 |
| Aortic Valve Surgery | 140 | (35.3%) | 142 | (40.2%) | 280 | (34.9%) | 0.197 |
| Aortic Valve Surgery Type |  | | | | | | |
| Aortic Valve Repair | 23 | (17.2%) | 41 | (35.7%) | 49 | (20.3%) | 0.006 |
| Biological Prosthesis | 79 | (59.0%) | 55 | (47.8%) | 134 | (55.6%) |  |
| Mechanical Prosthesis | 32 | (23.9%) | 19 | (16.5%) | 58 | (24.1%) |  |
| Mitral Valve Surgery | 117 | (29.5%) | 130 | (36.8%) | 251 | (31.9%) | 0.091 |
| Mitral Valve Surgery Type |  | | | | | | |
| Mitral Valve Repair | 44 | (38.6%) | 53 | (46.5%) | 95 | (41.7%) | 0.537 |
| Biological Prosthesis | 42 | (36.8%) | 38 | (33.3%) | 71 | (31.1%) |  |
| Mechanical Prosthesis | 28 | (24.6%) | 23 | (20.2%) | 62 | (13.0%) |  |
| Tricuspid Valve Surgery | 51 | (12.8%) | 62 | (17.6%) | 104 | (13.0%) | 0.086 |
| Aortic Surgery | 84 | (21.2%) | 78 | (22.1%) | 167 | (20.8%) | 0.833 |
| Aortic Surgery Type |  | | | | | | |
| Aortic Root | 12 | (14.3%) | 13 | (17.1%) | 27 | (16.4%) | 0.245 |
| Ascending Aorta And Root | 23 | (27.4%) | 20 | (26.3%) | 50 | (30.5%) |  |
| Ascending Aorta | 24 | (28.6%) | 24 | (31.6%) | 34 | (20.6%) |  |
| Ascending Aorta And Arch | 24 | (28.6%) | 16 | (21.1%) | 40 | (24.2%) |  |
| Aortic Arch And Descending Aorta | 1 | (1.2%) | 3 | (3.9%) | 14 | (8.5%) |  |
| Left Ventricular Assist Device | *5* | (1.3%) | 5 | (1.4%) | 10 | (1.2%) | 0.971 |
| Right Ventricular Assist Device | 3 | (0.8%) | 1 | (0.3%) | 0 | (0.0%) | 0.052 |
| Heart Transplantation | 26 | (6.5%) | 42 | (11.9%) | 59 | (7.3%) | 0.013 |
| Off-Pump Surgery | 14 | (3.6%) | 7 | (2.0%) | 48 | (6.0%) | 0.006 |
| Conversion To Cardiopulmonary Bypass | 6 | (42.9%) | 4 | (57.1%) | 11 | (21.6%) | 0.069 |
| Cardiopulmonary Bypass Time (min) | 215 | (146-290) | 235 | (169-312) | 189 | (125-267) | <0.001 |
| Crossclamp Time (min) | 108 | (69-155) | 108 | (72-159) | 99 | (64-148) | 0.045 |
| Intraoperative Transfusions | 182 | (91.0%) | 50 | (100.0%) | 403 | (92.0%) | 0.096 |
| Data are reported as n (% as valid percentage excluding missing values) or median (interquartile range). | | | | | | | |

**Supplementary Table 7.** Details On Extracorporeal Life Support of the Population 2010-2020

|  | **Aortic (n=397)** | | **Subclavian/Axillary (n=353)** | | **Femoral (n=803)** | | **P-value** |
| --- | --- | --- | --- | --- | --- | --- | --- |
| ECLS Indication |  | | | | | | |
| Failure to Wean | 146 | (37.2%) | 179 | (53.8%) | 242 | (30.3%) | <0.001 |
| Acute Pulmonary Embolism | 0 | (0.0%) | 0 | (0.0%) | 2 | (0.3%) |  |
| Arrhythmia | 8 | (2.0%) | 4 | (1.2%) | 21 | (2.6%) |  |
| Cardiac Arrest | 39 | (9.9%) | 10 | (3.0%) | 81 | (10.2%) |  |
| Cardiogenic Shock | 107 | (27.2%) | 57 | (17.1%) | 252 | (31.6%) |  |
| Pulmonary Hemorrhage | 3 | (0.8%) | 1 | (0.3%) | 2 | (0.3%) |  |
| Right Ventricular Failure | 51 | (13.0%) | 38 | (11.4%) | 98 | (12.3%) |  |
| Respiratory Failure | 6 | (1.5%) | 20 | (6.0%) | 12 | (1.5%) |  |
| Biventricular Failure | 28 | (7.1%) | 20 | (6.0%) | 72 | (9.0%) |  |
| Other | 5 | (1.3%) | 4 | (1.2%) | 16 | (2.0%) |  |
| Left Ventricle Unloading | 150 | (45.5%) | 34 | (13.1%) | 218 | (31.1%) | <0.001 |
| ECLS Duration (hours) | 120 | (59-216) | 118 | (70-187) | 120 | (54-192) | 0.570 |
| Anticoagulation Regimen |  | | | | | | |
| None | 48 | (12.3%) | 19 | (5.7%) | 65 | (8.3%) | 0.004 |
| Heparin | 338 | (86.7%) | 316 | (94.0%) | 709 | (90.8%) |  |
| Bivalirudin | 3 | (0.8%) | 0 | (0.0%) | 0 | (0.0%) |  |
| Argatroban | 0 | (0.0%) | 0 | (0.0%) | 3 | (0.4%) |  |
| Data are reported as n (% as valid percentage excluding missing values) or median (interquartile range). ECLS: Extracorporeal Life Support. | | | | | | | |

**Supplementary Table 8.** Post-Operative Outcomes of the Population 2010-2020

|  | **Aortic (n=397)** | | **Subclavian/ Axillary (n=353)** | | **Femoral (n=803)** | | **P-value** |
| --- | --- | --- | --- | --- | --- | --- | --- |
| Intensive Care Unit Stay (days) | 12 | (5-25) | 16 | (8-33) | 13 | (6-24) | <0.001 |
| Hospital Stay (days) | 17 | (6-35) | 24 | (10-46) | 20 | (8-39) | <0.001 |
| Postoperative Bleeding | 254 | (65.5%) | 186 | (53.0%) | 426 | (53.9%) | <0.001 |
| Requiring Rethoracotomy | 166 | (43.0%) | 116 | (42.5%) | 282 | (35.6%) | 0.020 |
| Cannulation Site Bleeding | 44 | (11.4%) | 51 | (14.7%) | 84 | (10.6%) | 0.143 |
| Diffuse No-Surgical Related Bleeding | 113 | (29.2%) | 73 | (25.7%) | 145 | (19.8%) | 0.001 |
| Composite End-Point Of Neurological Complications | 57 | (14.4%) | 68 | (19.3%) | 99 | (12.5%) | 0.011 |
| Brain Edema | 12 | (3.0%) | 18 | (5.3%) | 25 | (3.4%) | 0.217 |
| Cerebral Hemorrhage | 9 | (2.3%) | 20 | (6.0%) | 17 | (2.3%) | 0.003 |
| Severity: |  | | | | | | |
| Minor | 4 | (44.4%) | 7 | (46.7%) | 4 | (27.3%) | 0.099 |
| Disabling | 2 | (22.2%) | 7 | (46.7%) | 3 | (27.3%) |  |
| Fatal | 3 | (33.3%) | 1 | (6.7%) | 6 | (54.5%) |  |
| Seizure | 4 | (1.0%) | 12 | (3.5%) | 10 | (1.4%) | 0.018 |
| Stroke | 34 | (10.9%) | 56 | (15.9%) | 73 | (9.2%) | 0.004 |
| Severity: |  | | | | | | |
| Minor | 14 | (36.8%) | 27 | (61.4%) | 20 | (33.3%) | 0.041 |
| Disabling | 15 | (39.5%) | 11 | (25.0%) | 21 | (35.0%) |  |
| Fatal | 9 | (23.7%) | 6 | (13.6%) | 19 | (31.7%) |  |
| Leg Ischemia | 24 | (6.3%) | 16 | (4.6%) | 96 | (13.0%) | <0.001 |
| Cardiac Arrest | 60 | (15.2%) | 48 | (16.7%) | 119 | (16.1%) | 0.864 |
| Bowel Ischemia | 23 | (5.8%) | 22 | (7.6%) | 42 | (5.7%) | 0.491 |
| Right Ventricular Failure | 80 | (20.3%) | 86 | (34.1%) | 118 | (16.0%) | <0.001 |
| Acute Kidney Injury | 198 | (50.1%) | 203 | (71.2%) | 360 | (48.7%) | <0.001 |
| Pneumonia | 84 | (21.3%) | 80 | (31.7%) | 153 | (20.7%) | 0.001 |
| Septic Shock | 69 | (17.5%) | 42 | (16.7%) | 127 | (17.2%) | 0.968 |
| Vasoplegic Syndrome | 45 | (11.4%) | 23 | (9.2%) | 91 | (12.3%) | 0.406 |
| Acute Respiratory Distress Syndrome | 15 | (3.8%) | 20 | (6.9%) | 27 | (3.7%) | 0.057 |
| Multiple Organ Failure | 159 | (40.3%) | 114 | (32.8%) | 256 | (32.0%) | 0.015 |
| Vascular Surgery | 26 | (6.6%) | 23 | (9.3%) | 108 | (14.6%) | <0.001 |
| In-Hospital Mortality | 274 | (69.0%) | 195 | (55.2%) | 477 | (59.4%) | <0.001 |
| Main Cause of Death |  | | | | | | |
| Multiorgan Failure | 92 | (38.2%) | 72 | (39.1%) | 178 | (39.4%) | 0.289 |
| Sepsis | 13 | (5.4%) | 17 | (9.2%) | 33 | (7.3%) |  |
| Persistent Heart Failure | 90 | (37.3%) | 67 | (36.4%) | 152 | (33.6%) |  |
| Vasoplegia | 7 | (2.9%) | 1 | (0.5%) | 9 | (2.0%) |  |
| Bleeding | 18 | (7.5%) | 6 | (3.3%) | 25 | (5.5%) |  |
| Neurological Injury | 8 | (3.3%) | 12 | (6.5%) | 24 | (5.3%) |  |
| Bowel Ischemia | 7 | (2.9%) | 1 | (0.5%) | 10 | (2.2%) |  |
| Other | 6 | (2.5%) | 8 | (4.3%) | 21 | (4.6%) |  |
| Data are reported as n (% as valid percentage excluding missing values) or median (interquartile range). | | | | | | | |

**Sensitivity Analysis after Excluding Patients who Suffered Previous Stroke, Previous Transient Ischemic Attack, Peripheral Vessel Disease or with Cardiac Arrest During Hospital Stay.**

**Supplementary Table 9.** Baseline Characteristic. Exclusion of Previous Stroke, Transient Ischemic Attack, Peripheral Vessel Disease or Cardiac Arrest

|  | **Aortic (n=410)** | | **Subclavian/Axillary (n=315)** | | **Femoral (n=846)** | | **P-value** |
| --- | --- | --- | --- | --- | --- | --- | --- |
| Age (years) | 64 | (53-71) | 67 | (56-74) | 64 | (55-71) | 0.011 |
| Sex |  | | | | | | |
| Female | 124 | (40.3%) | 102 | (48.3%) | 254 | (38.8%) | 0.049 |
| Male | 184 | (59.7%) | 109 | (51.7%) | 400 | (61.2%) |  |
| BSA (m^2^) | 1.87 | (1.71-2.03) | 1.94 | (1.80-2.07) | 1.87 | (1.72-2.03) | 0.002 |
| Comorbidities |  | | | | | | |
| Hypertension | 173 | (56.2%) | 146 | (69.2%) | 384 | (58.8%) | 0.008 |
| Dialysis | 17 | (5.2%) | 22 | (11.4%) | 48 | (7.4%) | 0.061 |
| Previous Myocardial Infarction | 78 | (25.3%) | 72 | (34.1%) | 158 | (24.2%) | 0.015 |
| Previous Endocarditis | 22 | (7.1%) | 23 | (10.9%) | 40 | (6.1%) | 0.066 |
| Smoking | 65 | (21.7%) | 63 | (38.7%) | 144 | (24.4%) | <0.001 |
| Atrial Fibrillation | 88 | (28.6%) | 63 | (30.0%) | 165 | (25.2%) | 0.303 |
| Previous Pulmonary Embolism | 6 | (1.9%) | 8 | (5.0%) | 6 | (0.9%) | 0.002 |
| Diabetes Mellitus | 67 | (21.8%) | 55 | (26.1%) | 138 | (21.1%) | 0.303 |
| COPD | 22 | (7.1%) | 34 | (18.8%) | 62 | (9.6%) | <0.001 |
| Left Ventricle Ejection Fraction (%) | 47 | (30-59) | 40 | (25-60) | 45 | (30-60) | 0.107 |
| Euroscore II | 7.9 | (3.3-16.7) | 14.0 | (6.3-26.0) | 5.4 | (2.4-16.0) | <0.001 |
| Preoperative Condition | | | | | | | |
| NYHA Class |  | | | | | | |
| Class I | 18 | (6.3%) | 15 | (7.1%) | 46 | (7.3%) | <0.001 |
| Class II | 64 | (22.2%) | 32 | (15.2%) | 138 | (21.9%) |  |
| Class III | 110 | (38.2%) | 66 | (31.4%) | 273 | (43.4%) |  |
| Class IV | 96 | (33.3%) | 97 | (46.2%) | 172 | (27.3%) |  |
| Preoperative Cardiogenic Shock | 78 | (25.3%) | 46 | (23.1%) | 115 | (17.6%) | 0.015 |
| Preoperative Right Ventricular Failure | 35 | (11.7%) | 23 | (13.6%) | 61 | (9.8%) | 0.307 |
| Preoperative Biventricular Failure | 19 | (6.5%) | 18 | (12.9%) | 37 | (6.6%) | 0.031 |
| Emergency Surgery | 92 | (29.9%) | 66 | (32.8%) | 125 | (19.1%) | <0.001 |
| Data are reported as n (% as valid percentage excluding missing values) or median (interquartile range). BSA: Body Surface Area; COPD: Chronic Obstructive Pulmonary Disease; NYHA: New York Heart Association. | | | | | | | |

**Supplementary Table 10.** Procedural Characteristics. Exclusion of Previous Stroke, Transient Ischemic Attack, Peripheral Vessel Disease or Cardiac Arrest

|  | **Aortic (n=410)** | | **Subclavian/Axillary (n=315)** | | **Femoral (n=846)** | | **P-value** |
| --- | --- | --- | --- | --- | --- | --- | --- |
| Coronary Artery Bypass Graft | 117 | (38.0%) | 99 | (46.9%) | 261 | (39.9%) | 0.106 |
| Aortic Valve Surgery | 103 | (33.4%) | 80 | (37.9%) | 204 | (31.2%) | 0.192 |
| Aortic Valve Surgery Type |  | | | | | | |
| Aortic Valve Repair | 13 | (12.9%) | 21 | (31.3%) | 33 | (17.8%) | 0.038 |
| Biological Prosthesis | 65 | (64.4%) | 33 | (49.3%) | 104 | (56.2%) |  |
| Mechanical Prosthesis | 23 | (22.8%) | 13 | (19.4%) | 48 | (25.9%) |  |
| Mitral Valve Surgery | 104 | (33.8%) | 74 | (35.1%) | 200 | (30.6%) | 0.391 |
| Mitral Valve Surgery Type |  | | | | | | |
| Mitral Valve Repair | 39 | (38.6%) | 31 | (48.4%) | 72 | (37.7%) | 0.026 |
| Biological Prosthesis | 40 | (39.6%) | 23 | (35.9%) | 55 | (28.8%) |  |
| Mechanical Prosthesis | 22 | (21.8%) | 10 | (15.6%) | 64 | (33.5%) |  |
| Tricuspid Valve Surgery | 47 | (15.3%) | 41 | (19.4%) | 98 | (15.0%) | 0.290 |
| Aortic Surgery | 67 | (21.8%) | 43 | (20.4%) | 108 | (16.5%) | 0.114 |
| Aortic Surgery Type |  | | | | | | |
| Aortic Root | 7 | (10.4%) | 8 | (19.0%) | 17 | (15.9%) | 0.386 |
| Ascending Aorta And Root | 23 | (34.3%) | 12 | (28.6%) | 30 | (28.0%) |  |
| Ascending Aorta | 19 | (28.4%) | 13 | (31.0%) | 26 | (24.3%) |  |
| Ascending Aorta And Arch | 17 | (25.4%) | 9 | (21.4%) | 26 | (24.3%) |  |
| Aortic Arch And Descending Aorta | 1 | (1.5%) | 0 | (0.0%) | 8 | (7.5%) |  |
| LVAD | 5 | (1.6%) | 4 | (1.9%) | 6 | (0.9%) | 0.449 |
| RVAD | 4 | (1.3%) | 1 | (0.5%) | 0 | (0.0%) | 0.015 |
| Heart Transplantation | 28 | (9.1%) | 32 | (15.2%) | 76 | (11.6%) | 0.105 |
| Off-Pump Surgery | 6 | (2.0%) | 4 | (1.9%) | 35 | (5.4%) | 0.010 |
| Conversion to Cardiopulmonary Bypass | 3 | (50.0%) | 2 | (50.0%) | 7 | (18.9%) | 0.035 |
| Cardiopulmonary Bypass Time (min) | 225 | (153-309) | 216 | (153-297) | 200 | (132-288) | 0.009 |
| Crossclamp Time (min) | 112 | (63-165) | 102 | (67-168) | 99 | (64-153) | 0.073 |
| Intraoperative Transfusions | 146 | (90.1%) | 38 | (100.0%) | 339 | (92.6%) | 0.114 |
| Data are reported as n (% as valid percentage excluding missing values) or median (interquartile range). LVAD: Left Ventricular Assist Device; RVAD: Right Ventricular Assist Device. | | | | | | | |

**Supplementary Table 11.** Details on ECLS. Exclusion of Previous Stroke, Transient Ischemic Attack, Peripheral Vessel Disease or Cardiac Arrest

|  | **Aortic (n=410)** | | **Subclavian/Axillary (n=315)** | | **Femoral (n=846)** | | **P-value** |
| --- | --- | --- | --- | --- | --- | --- | --- |
| ECLS Indication |  | | | | | | |
| Failure to Wean | 132 | (42.9%) | 111 | (52.9%) | 235 | (35.9%) | <0.001 |
| Acute Pulmonary Embolism | 0 | (0.0%) | 0 | (0.0%) | 1 | (0.2%) |  |
| Arrhythmia | 5 | (1.6%) | 3 | (1.4%) | 20 | (3.1%) |  |
| Cardiogenic Shock | 89 | (28.9%) | 38 | (18.1%) | 214 | (32.7%) |  |
| Pulmonary Hemorrhage | 3 | (1.0%) | 1 | (0.5%) | 1 | (0.2%) |  |
| Right Ventricular Failure | 43 | (14.0%) | 33 | (15.7%) | 87 | (13.3%) |  |
| Respiratory Failure | 5 | (1.6%) | 13 | (6.2%) | 16 | (2.4%) |  |
| Biventricular Failure | 26 | (8.4%) | 9 | (4.3%) | 66 | (10.1%) |  |
| Other | 5 | (1.6%) | 2 | (1.0%) | 14 | (2.1%) |  |
| ECLS Duration (hours) | 120 | (69-211) | 116 | (73-178) | 120 | (65-195) | 0.823 |
| Anticoagulation Regimen |  | | | | | | |
| None | 34 | (11.30%) | 7 | (3.60%) | 50 | (7.80%) | 0.009 |
| Heparin | 263 | (87.4%) | 187 | (95.9%) | 581 | (91.2%) |  |
| Bivalirudin | 3 | (1.0%) | 0 | (0.0%) | 0 | (0.0%) |  |
| Argatroban | 0 | (0.0%) | 0 | (0.0%) | 3 | (0.5%) |  |
| Data are reported as n (% as valid percentage excluding missing values) or median (interquartile range). ECLS: Extracorporeal Life Support. | | | | | | | |

**Supplementary Table 12.** Post-operative outcomes. Exclusion of Previous Stroke, Transient Ischemic Attack, Peripheral Vessel Disease or Cardiac Arrest

|  | **Aortic (n=410)** | | **Subclavian/Axillary (n=315)** | | **Femoral (n=846)** | | **P-value** |
| --- | --- | --- | --- | --- | --- | --- | --- |
| Intensive Care Unit Stay (days) | 12 | (6-23) | 17 | (10-31) | 13 | (6-24) | <0.001 |
| Hospital Stay (days) | 17 | (7-34) | 28 | (14-49) | 21 | (9-42) | <0.001 |
| Postoperative Bleeding | 208 | (69,3%) | 101 | (48,1%) | 345 | (53.4%) | <0.001 |
| Requiring Rethoracotomy | 145 | (48.5%) | 60 | (35.7%) | 237 | (37.1%) | 0.002 |
| Cannulation Site Bleeding | 30 | (10.0%) | 27 | (13.0%) | 73 | (11.3%) | 0.587 |
| Diffuse No-Surgical Related Bleeding | 92 | (30.8%) | 46 | (25.4%) | 128 | (20.1%) | 0.001 |
| Composite End-Point Of Neurological Complications | 48 | (15.6%) | 38 | (18.0%) | 62 | (9.6%) | 0.001 |
| Brain Edema | 11 | (3.6%) | 9 | (4.4%) | 14 | (2.2%) | 0.183 |
| Cerebral Hemorrhage | 8 | (2.6%) | 12 | (5.9%) | 8 | (1.2%) | <0.001 |
| Severity: |  | | | | | | |
| Minor | 4 | (50.0%) | 4 | (44.4%) | 0 | (0.0%) | 0.048 |
| Disabling | 1 | (12.5%) | 4 | (44.4%) | 1 | (16.7%) |  |
| Fatal | 3 | (37.5%) | 1 | (11.1%) | 5 | (83.3%) |  |
| Seizure | 5 | (1.6%) | 3 | (1.5%) | 9 | (1.4%) | 0.959 |
| Stroke | 35 | (11.4%) | 33 | (15.6%) | 46 | (7.1%) | <0.001 |
| Severity: |  | | | | | | |
| Minor | 16 | (50.0%) | 15 | (62.5%) | 16 | (40.0%) | 0.491 |
| Disabling | 9 | (28.1%) | 6 | (25.0%) | 13 | (32.5%) |  |
| Fatal | 7 | (21.9%) | 3 | (12.5%) | 11 | (27.5%) |  |
| Leg Ischemia | 13 | (4.4%) | 7 | (3.4%) | 85 | (13.1%) | <0.001 |
| Bowel Ischemia | 16 | (5.2%) | 16 | (9.2%) | 30 | (4.7%) | 0.064 |
| Right Ventricular Failure | 55 | (17.9%) | 57 | (35.6%) | 114 | (17.7%) | <0.001 |
| Acute Kidney Injury | 164 | (53.6%) | 110 | (64.7%) | 338 | (52.6%) | 0.017 |
| Pneumonia | 70 | (22.8%) | 59 | (37.3%) | 123 | (19.1%) | <0.001 |
| Septic Shock | 56 | (18.2%) | 35 | (22.2%) | 106 | (16.5%) | 0.241 |
| Vasoplegic Syndrome | 32 | (10.4%) | 9 | (5.7%) | 81 | (12.6%) | 0.042 |
| Acute Respiratory Distress Syndrome | 11 | (3.6%) | 13 | (7.5%) | 26 | (4.0%) | 0.010 |
| Multiple Organ Failure | 130 | (42.3%) | 62 | (30.4%) | 200 | (31.1%) | 0.001 |
| Vascular Surgery | 20 | (6.5%) | 13 | (9.0%) | 79 | (12.5%) | 0.017 |
| In-Hospital Mortality | 215 | (69.8%) | 103 | (48.8%) | 370 | (56.6%) | <0.001 |
| Main Cause Of Death |  | | | | | | |
| Multiorgan Failure | 76 | (40.0%) | 39 | (41.5%) | 138 | (39.3%) | 0.588 |
| Sepsis | 15 | (7.9%) | 9 | (9.6%) | 28 | (8.0%) |  |
| Persistent Heart Failure | 67 | (35.3%) | 34 | (36.2%) | 123 | (35.0%) |  |
| Vasoplegia | 5 | (2.6%) | 0 | (0.0%) | 7 | (2.0%) |  |
| Bleeding | 11 | (5.8%) | 3 | (3.2%) | 24 | (6.8%) |  |
| Neurological Injury | 7 | (3.7%) | 8 | (8.5%) | 13 | (3.7%) |  |
| Bowel Ischemia | 4 | (2.1%) | 1 | (1.1%) | 6 | (1.7%) |  |
| Other | 5 | (2.6%) | 0 | (0.0%) | 12 | (3.4%) |  |
| Data are reported as n (% as valid percentage excluding missing values) or median (interquartile range). | | | | | | | |

**Supplementary Figures**

**Supplementary Figure 1**: Data Flow from PELS Database to Current Analysis.


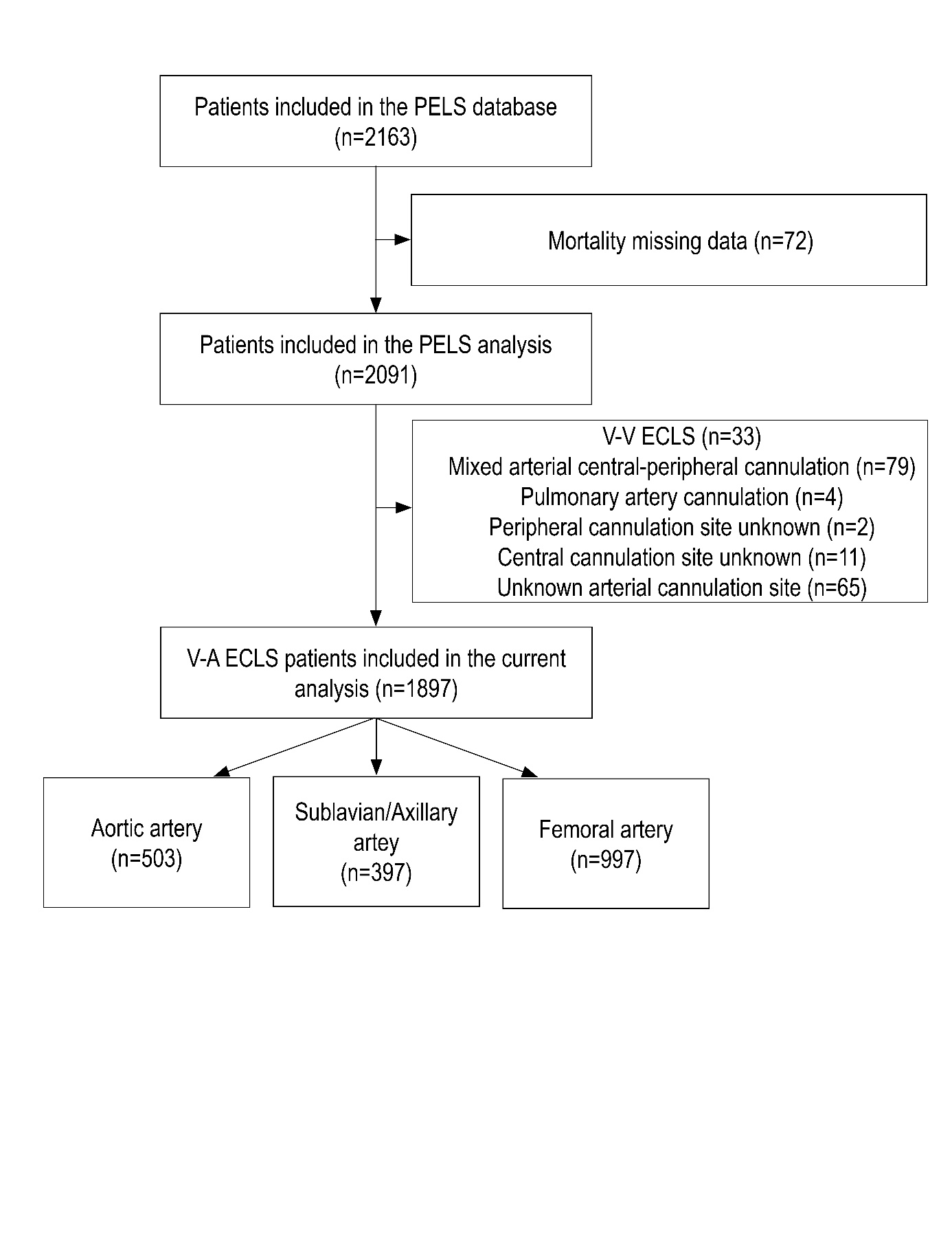


**Supplementary Figure 2:** Scatter plot representing the yearly contribution of patients from each center taking part in the PELS-1 study. The size of each point represents the number of patients added to the PELS-1 database in that year.


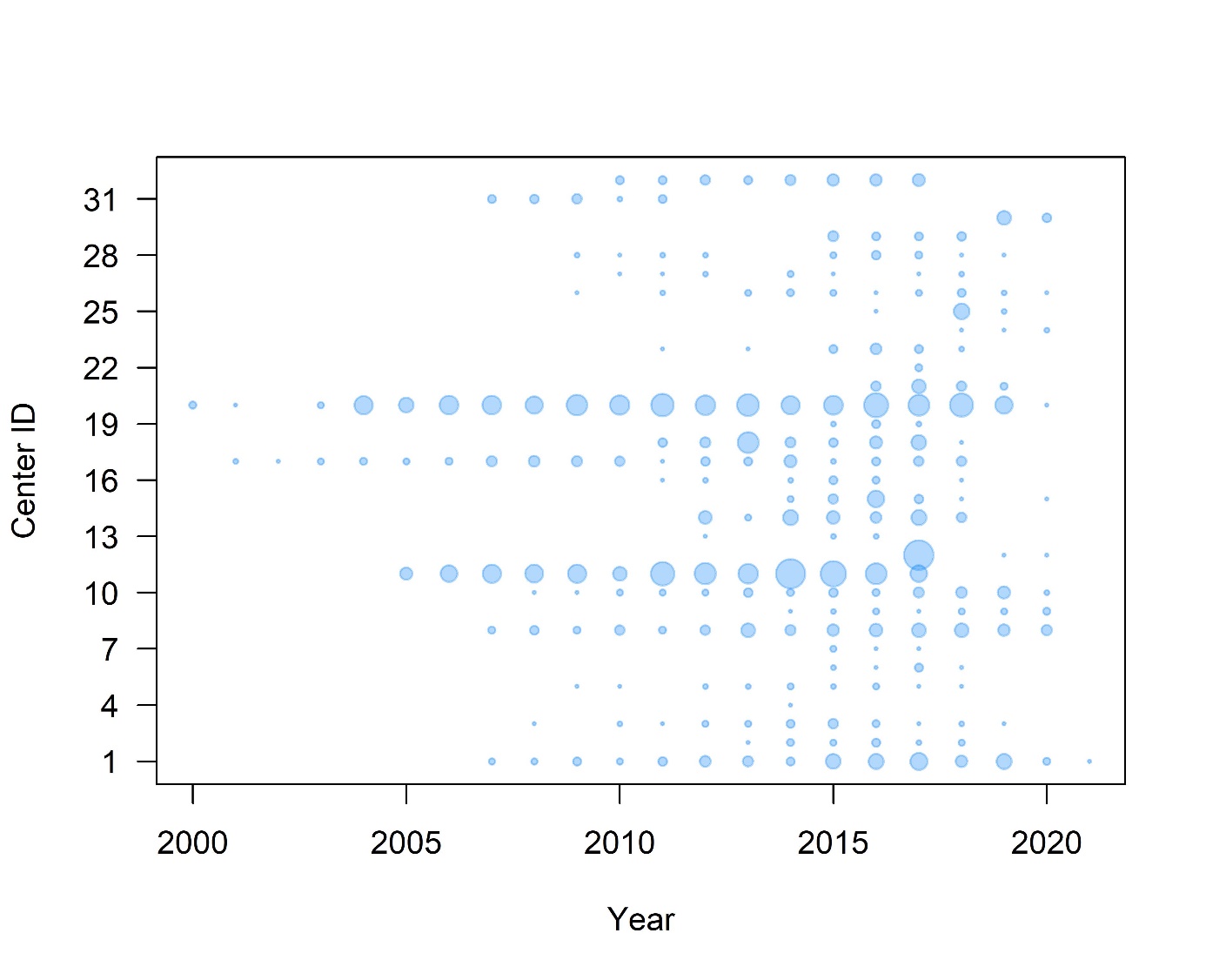


**Supplementary Figure 3**: Composite End-Point of Neurological Outcome in Sensitivity Analysis Including Patients From 2010 to 2020


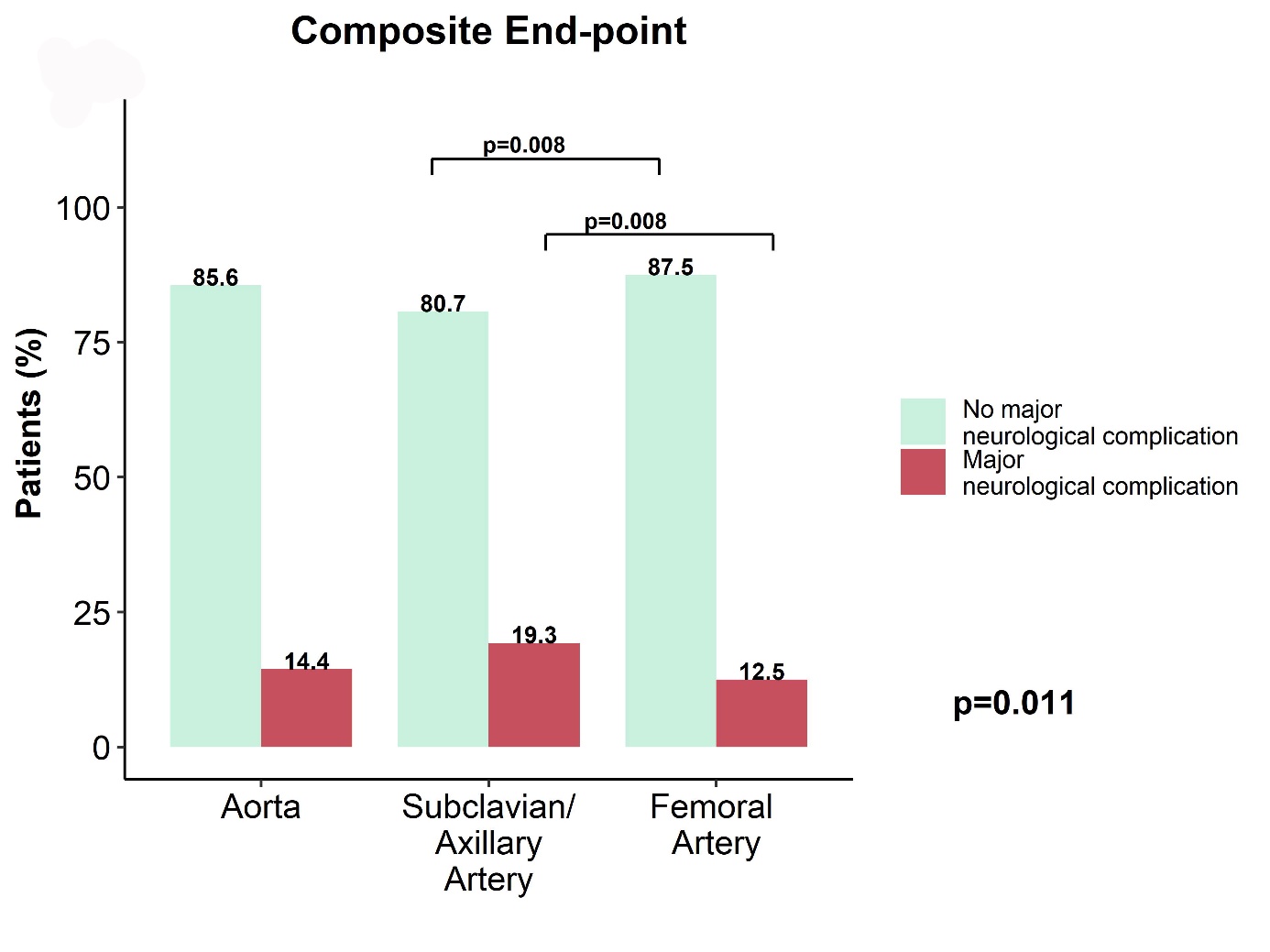


**Supplementary Figure 4:** Time of death in Sensitivity Analysis Including Patients From 2010 to 2020

**
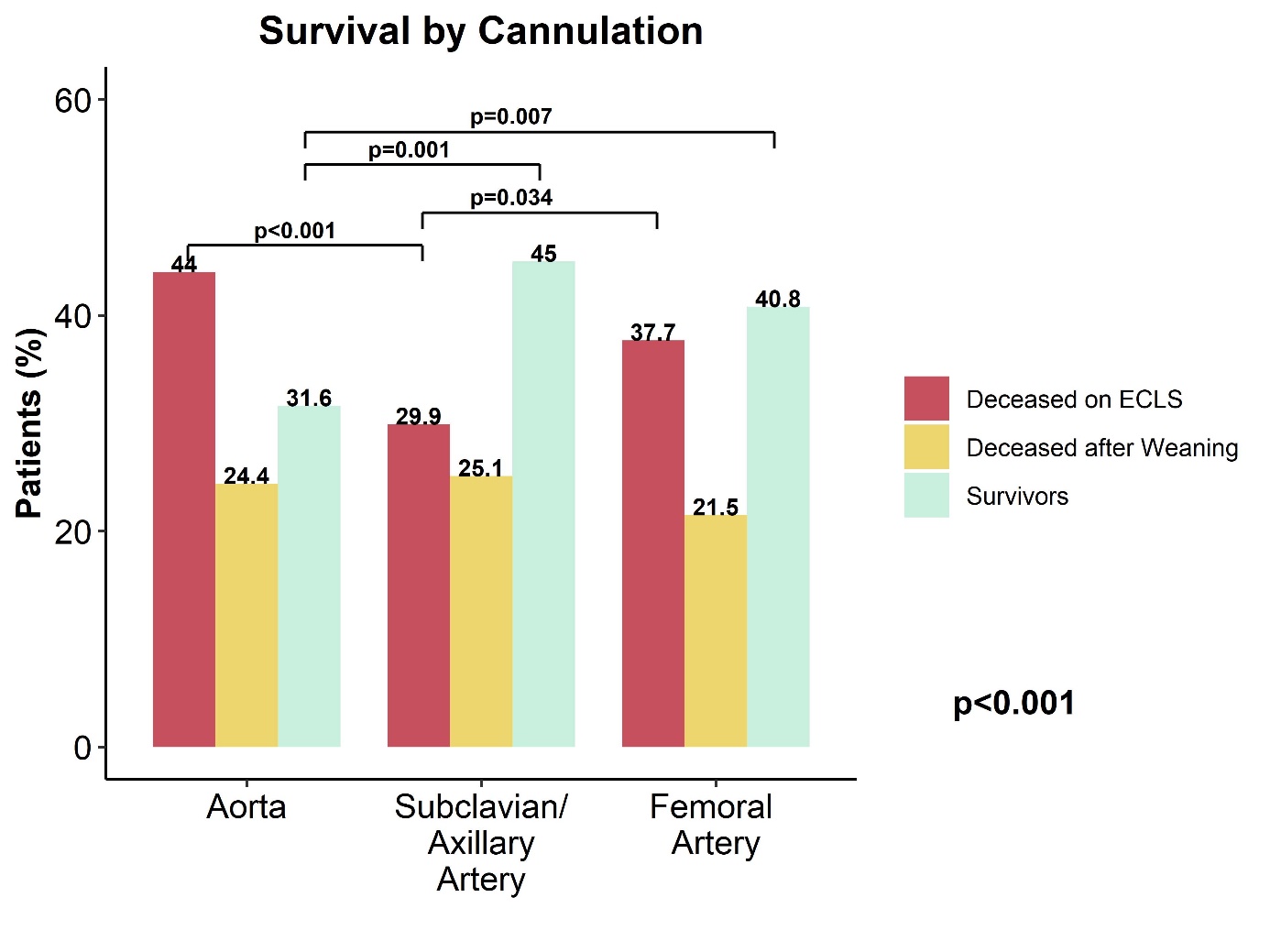
**

**Supplementary Figure 5**: Survival in Subclavian vs Femoral vs Aortic Cannulation including patients from 2010 to 2020


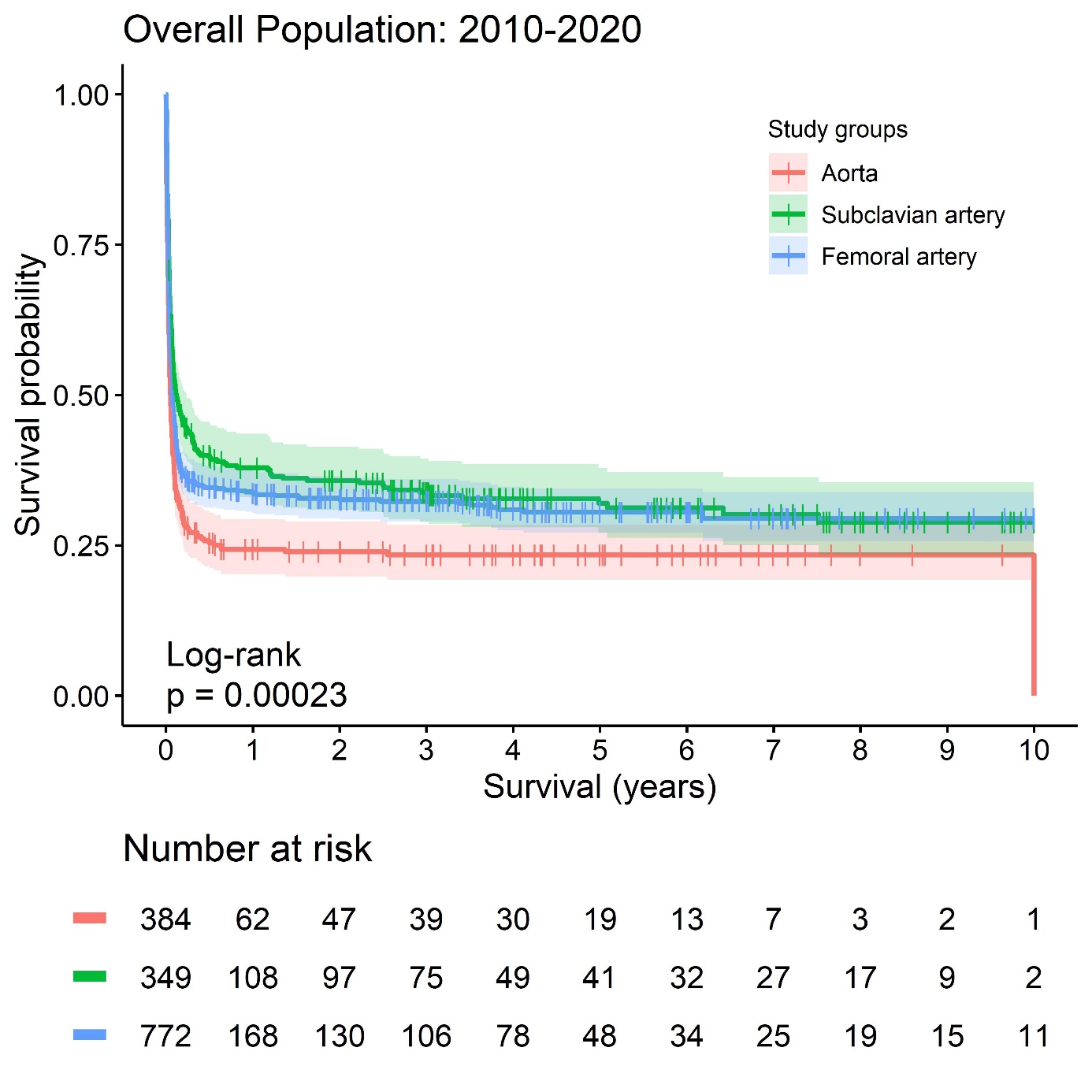


**Supplementary Figure 6**: Neurological Outcome Excluding Patients with Previous Stroke, Transient Ischemic Attack, Peripheral Vessel Disease or Cardiac Arrest


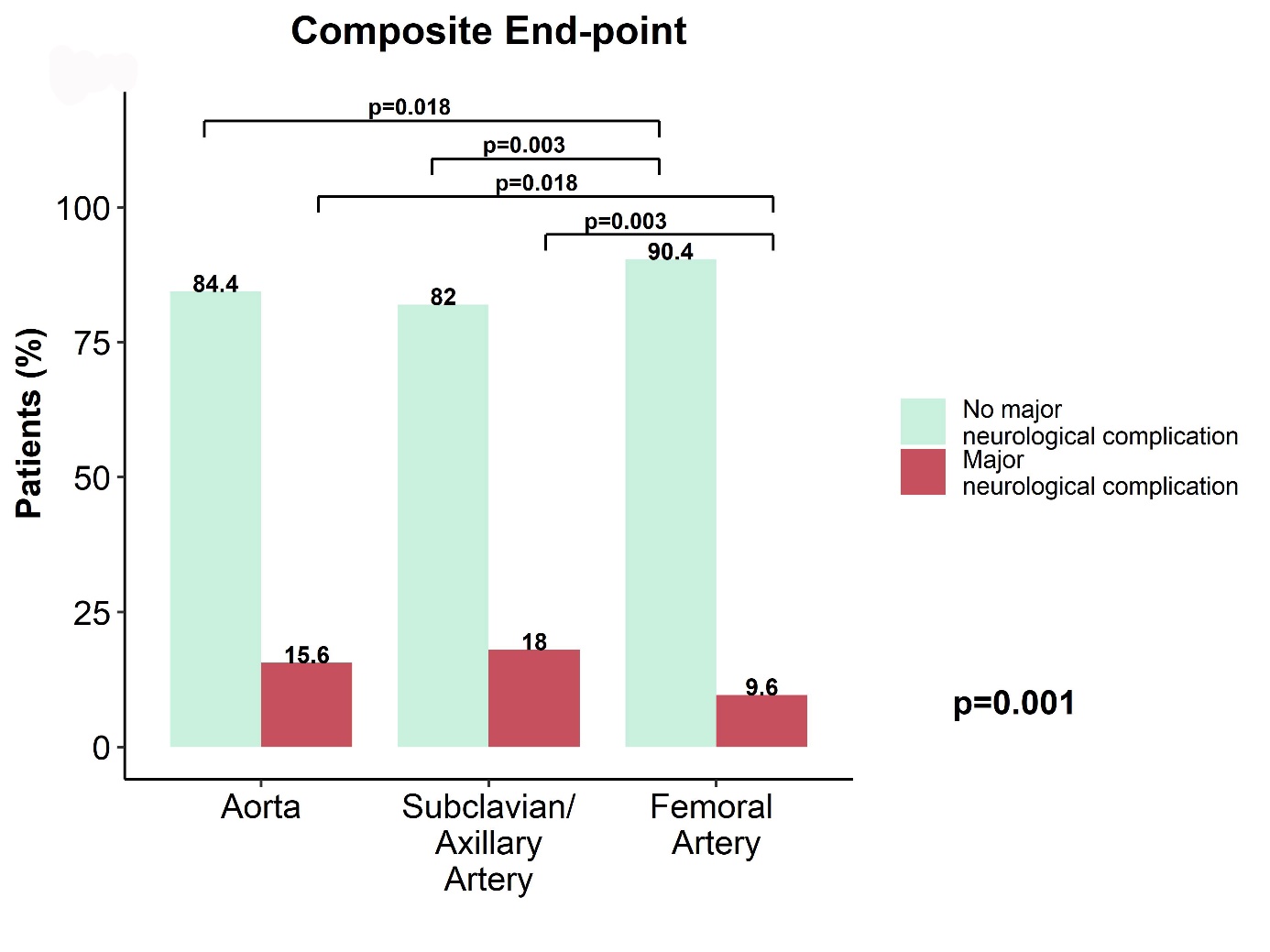


**Supplementary Figure 7**: Time of Death Excluding Previous Stroke, Transient Ischemic Attack, Peripheral Vessel Disease or Cardiac Arrest


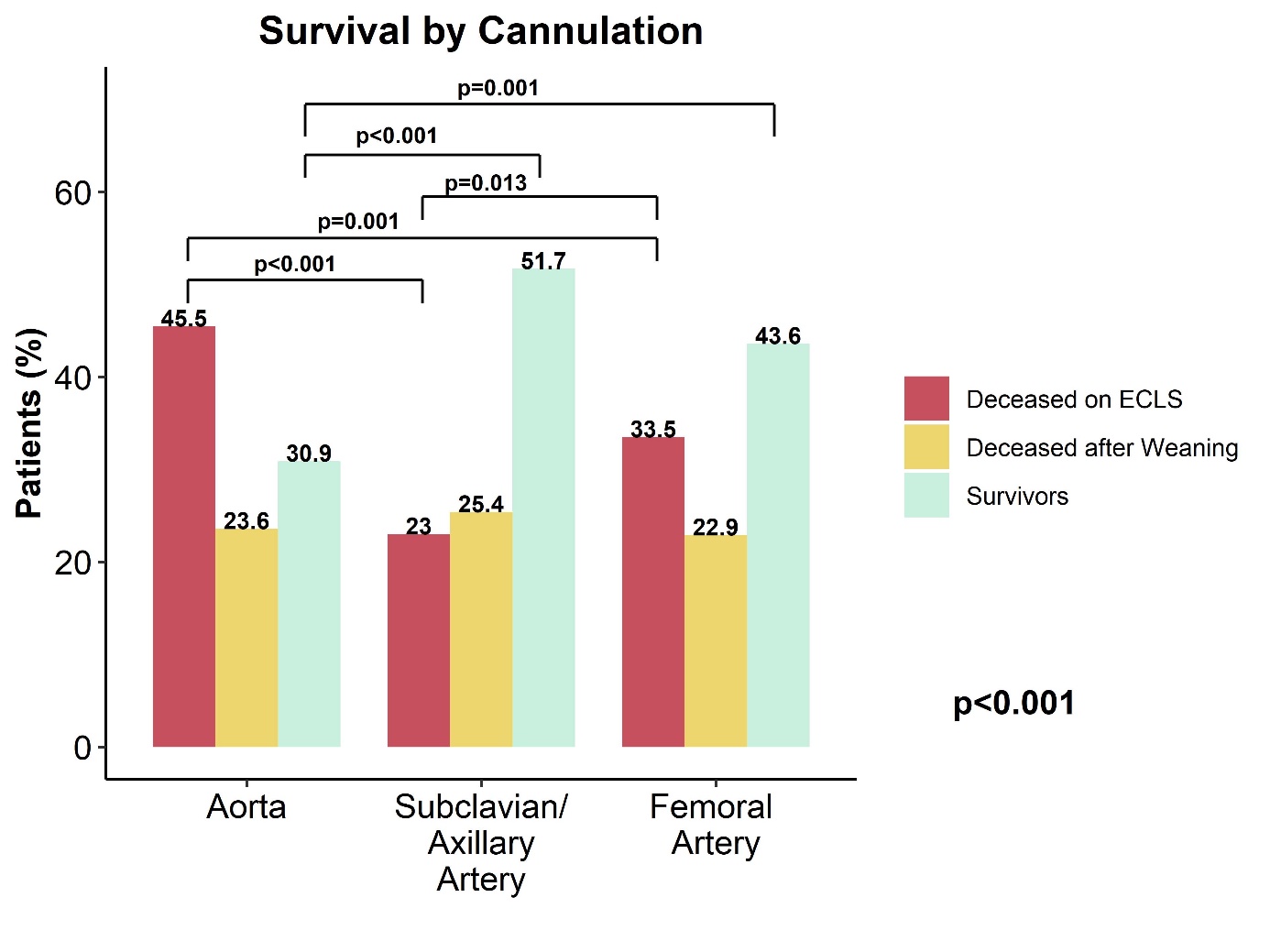


**Supplementary Figure 8**: Survival after Exclusion of Previous Stroke, Transient Ischemic Attack, Peripheral Vessel Disease or Cardiac Arrest.


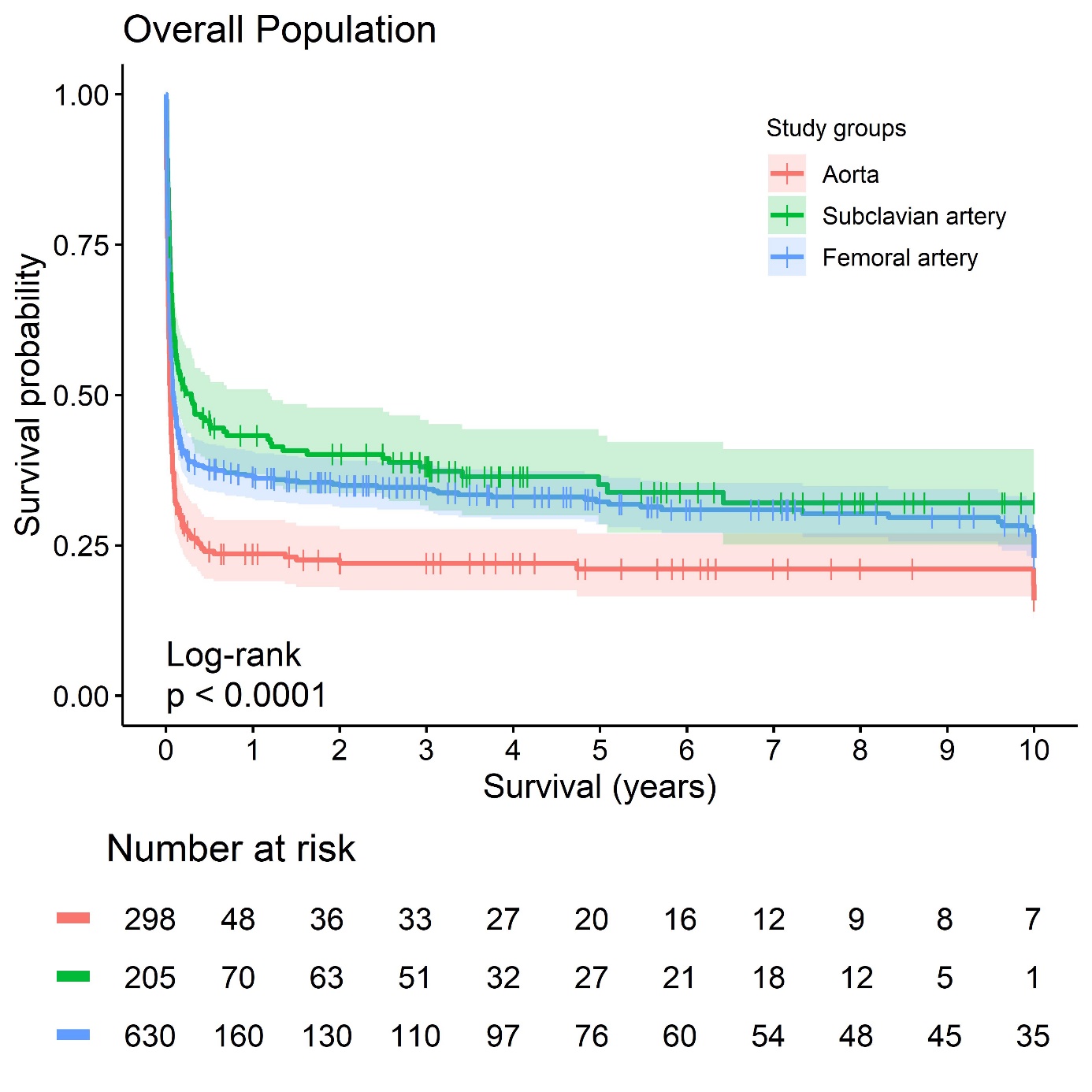


**References**

1. Lorusso R, Gelsomino S, Parise O, et al. Neurologic Injury in Adults Supported With Veno-Venous Extracorporeal Membrane Oxygenation for Respiratory Failure: Findings From the Extracorporeal Life Support Organization Database. *Crit Care Med*. Aug 2017;45(8):1389-1397. doi:10.1097/ccm.0000000000002502

2. Biancari F, Dalén M, Fiore A, et al. Multicenter study on postcardiotomy venoarterial extracorporeal membrane oxygenation. *J Thorac Cardiovasc Surg*. May 2020;159(5):1844-1854.e6. doi:10.1016/j.jtcvs.2019.06.039

3. Khanduja S, Kim J, Kang JK, et al. Hypoxic-Ischemic Brain Injury in ECMO: Pathophysiology, Neuromonitoring, and Therapeutic Opportunities. *Cells*. Jun 5 2023;12(11)doi:10.3390/cells12111546

4. Mateen FJ, Muralidharan R, Shinohara RT, Parisi JE, Schears GJ, Wijdicks EF. Neurological injury in adults treated with extracorporeal membrane oxygenation. *Arch Neurol*. Dec 2011;68(12):1543-9. doi:10.1001/archneurol.2011.209

5. Singer M, Deutschman CS, Seymour CW, et al. The Third International Consensus Definitions for Sepsis and Septic Shock (Sepsis-3). *JAMA*. Feb 23 2016;315(8):801-10. doi:10.1001/jama.2016.0287

6. Kowalewski M, Zieliński K, Brodie D, et al. Venoarterial Extracorporeal Membrane Oxygenation for Postcardiotomy Shock-Analysis of the Extracorporeal Life Support Organization Registry. *Crit Care Med*. Jul 1 2021;49(7):1107-1117. doi:10.1097/ccm.0000000000004922
